# De novo and recessive forms of congenital heart disease have distinct genetic and phenotypic landscapes

**DOI:** 10.1038/s41467-019-12582-y

**Published:** 2019-10-17

**Authors:** W. Scott Watkins, E. Javier Hernandez, Sergiusz Wesolowski, Brent W. Bisgrove, Ryan T. Sunderland, Edwin Lin, Gordon Lemmon, Bradley L. Demarest, Thomas A. Miller, Daniel Bernstein, Martina Brueckner, Wendy K. Chung, Bruce D. Gelb, Elizabeth Goldmuntz, Jane W. Newburger, Christine E. Seidman, Yufeng Shen, H. Joseph Yost, Mark Yandell, Martin Tristani-Firouzi

**Affiliations:** 1 Department of Human Genetics, University of Utah, Salt Lake City, UT 84112 USA; 2 USTAR Center for Genetic Discovery, University of Utah, Salt Lake City, UT 84112 USA; 3 Molecular Medicine Program, University of Utah, Salt Lake City, UT 84112 USA; 4 Division of Pediatric Cardiology, University of Utah School of Medicine, Salt Lake City, UT 83113 USA; 5 Department of Pediatrics, Stanford University School of Medicine, Palo Alto, CA 94305 USA; 6 Department of Pediatrics, Yale University School of Medicine, New Haven, CT 06416 USA; 7 Department of Genetics, Yale University School of Medicine, New Haven, CT 06416 USA; 8 Departments of Pediatrics and Medicine, Columbia University, NY, Columbia, NY 10032 USA; 9 Mindich Child Health and Development Institute, Departments of Pediatrics and Genetics & Genomic Sciences, Icahn School of Medicine at Mount Sinai, New York, NY 10029 USA; 10 Division of Cardiology, Department of Pediatrics, Children’s Hospital of Philadelphia, Philadelphia, PA 19104 USA; 11 Department of Cardiology, Boston Children’s Hospital, Boston, MA 02115 USA; 12 Departments of Genetics and Medicine, Harvard Medical School, Boston, 02115 USA; 13 Howard Hughes Medical Institute, Harvard Medical School, Boston, 02115 USA; 14 Departments of Systems Biology and Biomedical Informatics, Columbia University, New York, NY 10032 USA; 15 Nora Eccles Harrison Cardiovascular Research and Training Institute, and Division of Pediatric Cardiology, University of Utah, Salt Lake City, UT 84112 USA

**Keywords:** Computational biology and bioinformatics, Next-generation sequencing, Cardiovascular genetics, Congenital heart defects

## Abstract

The genetic architecture of sporadic congenital heart disease (CHD) is characterized by enrichment in damaging de novo variants in chromatin-modifying genes. To test the hypothesis that gene pathways contributing to de novo forms of CHD are distinct from those for recessive forms, we analyze 2391 whole-exome trios from the Pediatric Cardiac Genomics Consortium. We deploy a permutation-based gene-burden analysis to identify damaging recessive and compound heterozygous genotypes and disease genes, controlling for confounding effects, such as background mutation rate and ancestry. Cilia-related genes are significantly enriched for damaging rare recessive genotypes, but comparatively depleted for de novo variants. The opposite trend is observed for chromatin-modifying genes. Other cardiac developmental gene classes have less stratification by mode of inheritance than cilia and chromatin-modifying gene classes. Our analyses reveal dominant and recessive CHD are associated with distinct gene functions, with cilia-related genes providing a reservoir of rare segregating variation leading to CHD.

## Abstract (web summary)

Large whole-exome sequencing studies have suggested that the genetic architecture of syndromic congenital heart disease (CHD) is different from sporadic forms. Here, Watkins et al. estimate the relative contribution of damaging recessive and de novo genotypes to CHD in 2391 trios and find them to be associated with different gene functions.

## Introduction

Traditional case–control analyses (e.g. Genome-Wide Association Studies, GWAS) identify rare and common genetic variants associated with common disorders, with most variants exerting a small impact on the phenotype^1^. At the opposite extreme are classical Mendelian diseases such as neurofibromatosis type 1 and Huntington’s disease (dominantly inherited) or Tay-Sachs and cystic fibrosis (recessively inherited) that are typically characterized by high impact, highly penetrant damaging variants located in one or a few genes. Located somewhere between these two extremes are complex disorders such as autism spectrum disorder and sporadic congenital heart disease (CHD). While these diseases are also common, large-scale whole-exome (WES)- and whole-genome sequencing (WGS)-based analyses have defined the contribution of high-impact rare variants located in many different loci, with no one gene having a large population attributable risk (PAR)^2–7^. Thus, large-scale WES and WGS collections provide the necessary resolution and allelic representation to discover and quantify the impact of disease-causing variants for disorders with high allelic and locus heterogeneity^8–10^.

Large-scale WES cohorts have revealed unique features of the genetic architecture of CHD. For example, damaging de novo variants account for ~8% of CHD cases, but up to ~28% of cases associated with neurodevelopmental delay and extra-cardiac anomalies^3–5^. Likewise, a distinct genetic architecture for syndromic vs. sporadic CHD was reported, with unique enrichment in loss-of-function de novo and incompletely penetrant inherited genetic variants, respectively^6^. De novo variants associated with CHD are highly enriched in genes related to chromatin regulation^3–5^. More recently, support for the contribution of recessive genotypes to CHD was reported in a study of 2645 parent–offspring trios ascertained by the Pediatric Cardiac Genomics Consortium (PCGC). There, we noted enrichment in damaging recessive genotypes for the CHD subgroup associated with laterality clinical phenotypes and implicated variants in cilia-related genes as candidates for isolated human CHD^3^. Recessive, likely disease-causing, genotypes were also identified in cilia-related genes in a WES analysis of 323 CHD probands with laterality phenotypes^7^. Together, these observations are consistent with the report of an abundance of recessive mutations in genes related to cilia structure and regulation identified in a murine forward genetic screen^11^. However, the relative importance of de novo vs. recessive genotypes, and the relative contributions of chromatin regulatory genes compared to cilia-related genes within the genetic landscape of CHD, remains unclear.

In principle, large WES datasets make it possible to associate classes of genes with particular phenotypes and outcomes, and to measure the strength of those associations, so as to discover and describe the large-scale genetic and phenotypic landscapes of a complex disease via a process similar to category-wide association testing, or CWAS^2^. Toward this end, we analyze 2391 trios from the PCGC, using a methodology that allows us to identify additional gene-damaging compound recessive genotypes and new disease genes, while at the same time controlling for the confounding effects of ancestry, sequencing methodologies, and differences in genetic burden between genes and across functional classes of genes. Using these data, we measure the relative contribution of recessive and de novo genotypes to CHD, quantify the strength of their associations with cilia-related and chromatin-related genes, and estimate the magnitude of those associations. Our examination of multiple gene classes, signaling pathways, expression, and developmental categories reveals that CHD is critically influenced by rare variants with high and moderate impact in chromatin- and cilia-related genes. Moreover, we find that dominant and recessive forms of CHD are associated with distinct gene functions. That is, cilia-related genes are significantly enriched for damaging rare recessive genotypes but comparatively depleted for de novo variants. The opposite trend is observed for chromatin-modifying genes. Consistent with these findings, laterality phenotypes are also significantly more common in probands with damaging recessive genotypes than de novo genotypes, a consequence of the distinct genetic architecture of recessive CHD. Collectively, our findings demonstrate that amid the considerable genetic and phenotypic heterogeneity of CHD, there exists a network of highly significant associations between genotypes, gene functions and phenotypes.

## Results

### Overview

We investigate the relative contribution and enrichment profiles of various genes, gene functions, pathways, expression patterns, and phenotypes to CHD. As the potential number of combinations of these categories is intractably large, we examine those with prior association with CHD or logical relationship to CHD etiology. We further evaluate the findings from the enrichment analysis using a Bayesian network to discover and rigorously describe a network of associations between genotypes, gene functions, expression, and phenotypes within a large CHD cohort.

### Data sets and gene lists

**Fig. 1:**
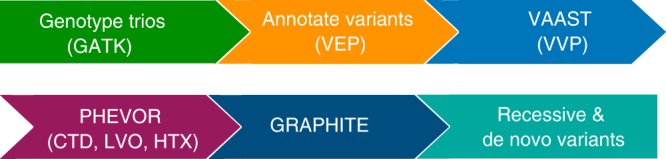
Candidate gene discovery pipeline. PCGC trios were joint-genotyped with the Genome Analysis Tool Kit (GATK). Variant calls were annotated with the Ensembl Variant Effect Predictor (VEP version 90). Each trio VCF file was processed with the Variant Annotation, Analysis and Search Tool (VAAST version 3). Every candidate gene identified by VAAST in each trio was re-ranked with the Phenotype Driven Variant Ontological Re-ranking Tool (PHEVOR) using human phenotype ontology (HPO) terms matching the proband’s phenotype. To assess variant quality and remove potential false positives, each variant was adjudicated with the graph-based alignment tool, GRAPHITE. A final list of all recessive and de novo candidate genes was assembled for 2391 probands

As a first step towards analyzing the genetic, functional, and phenotypic landscape of CHD, we used the Utah Genome Project’s FastQForward pipeline^12^ to analyze whole-exome trio data (proband, mother, and father) from the PCGC (Fig. 1). For the analyses reported here, we analyzed 2823 PCGC WES trios, 2391 of which passed our QC procedures (see Methods, Supplementary Fig. 1, and Supplementary Note 1).

**Fig. 2:**
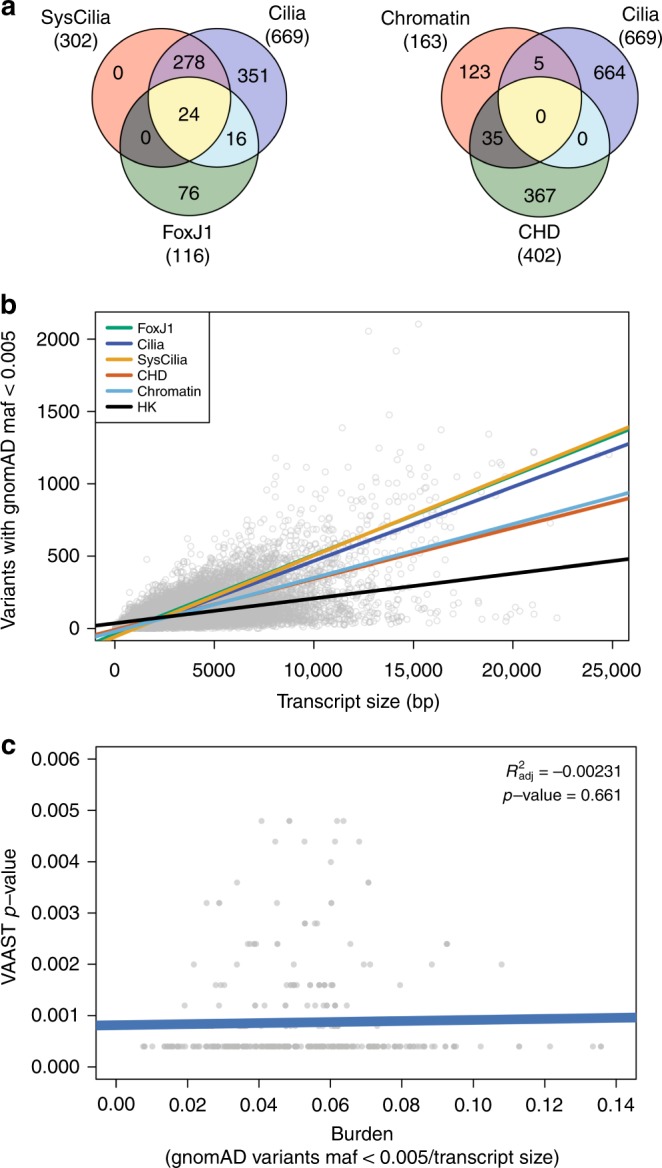
Cilia, chromatin, and CHD gene lists and burden analysis. **a** The relationships among five major gene sets used for enrichment analysis. Structural cilia (SysCilia) genes are a subset of all Cilia list genes. The CHD gene list has no overlap with cilia genes, and chromatin genes have only 3% overlap with cilia genes. **b** Gene burden by gene list. GnomAD-based gene-burden estimates (gray circles) are plotted as a function of transcript size. Regression lines for the genes contained in each major gene set are shown. The positive slopes indicate that, in general, large genes have higher burden than small genes. Cilia and FoxJ1-responsive genes show a higher rate (steeper slope) of increase in burden with gene size than do CHD or chromatin genes. Housekeeping genes have lower overall burden. **c** VAAST *p* values estimates are not affected by gene length or gene-burden. VAAST *p* values for damaged genes (gray dots) are plotted as a function of gene burden. The regression line (blue) shows the relationship between the VAAST *p* value estimates for damaged genes found in CHD probands and gene burden normalized by transcript size. No significant relationship between the number of damaged genes discovered nor magnitude of the *p* value as a function of gene burden is observed (coefficient of variation (*R*^2^_adj_) = −0.00231, *p* value ≥ 0.66, linear-model *F*-test)

To evaluate the contributions of cilia and cilia-related genes to CHD, we created three cilia gene lists (Fig. 2a, Supplementary Data 1): (1) a SysCilia gene list containing 302 well-characterized structural cilia genes^13^, (2) an expanded cilia gene list that contains 367 cilia-related genes plus all SysCilia genes, and (3) a FoxJ1 gene list containing 116 human homologs of zebrafish genes that are transcriptionally responsive to alterations in transcription factor FoxJ1a expression. FoxJ1 is a key transcriptional regulator of motile cilia genes. Additional genes and gene lists were used to quantify their relative contribution to CHD. These lists include (1) a set of non-cilia genes previously implicated in CHD^4,5,14–16^ (referred to as the CHD list), (2) a set of chromatin-modifying genes found to be disrupted in CHD patients^3–5^ and (3) several gene lists assembled from the reactome^17^ and other pathway databases representing candidate genes and pathways associated with CHD including Notch, TGF-β, non-cilia cytoskeletal, and receptor serine–threonine kinase genes. Only 5 of 163 (~3%) chromatin genes overlap with the cilia gene list. We note that the gene lists used here are non-exhaustive, but encompass many genes characteristic of, or ontologically linked to, each category or pathway. Genes utilized as negative controls for each enrichment test include: (1) uniformly expressed housekeeping genes^18^ and (2) a series of randomly selected gene lists created to have an equal amount of burden compared to each experimental list.

A key challenge for our enrichment analysis was controlling for the increased burden of rare variants (i.e., the background mutation rate) in cilia genes. As expected, large genes generally contain more rare variation than smaller genes, but different gene classes also have different rates of burden (Fig. 2b). The observation that housekeeping genes have the lowest rate of burden is likely the result of the increased purifying selection pressures on these essential genes, especially when the variation is damaging and inherited recessively.

The different burden associated with each gene list complicates cross-list comparisons. Variant-filtering based approaches, which are often used for variant prioritization, will recover candidate variants in proportion to the burden of a given gene class—leading to false associations, and overestimates of the relative contribution of that gene class to a particular disease phenotype^19^. To avoid this pitfall, we used the association testing tool VAAST which, by design, controls for gene-specific differences in burden, meaning that the recovery of candidate disease-causing variants is not biased towards high-burden genes^20^. This approach allows for an unbiased comparison of the number of damaging de novo and recessive genotypes identified in the different gene lists among CHD probands. Figure 2c shows that genotypes scored as damaging by VAAST are not over-represented in larger, more burden-rich genes in general (see also Supplementary Figs. 2 and 3, Supplementary Notes 2 and 3).

### Permutation analyses

**Fig. 3:**
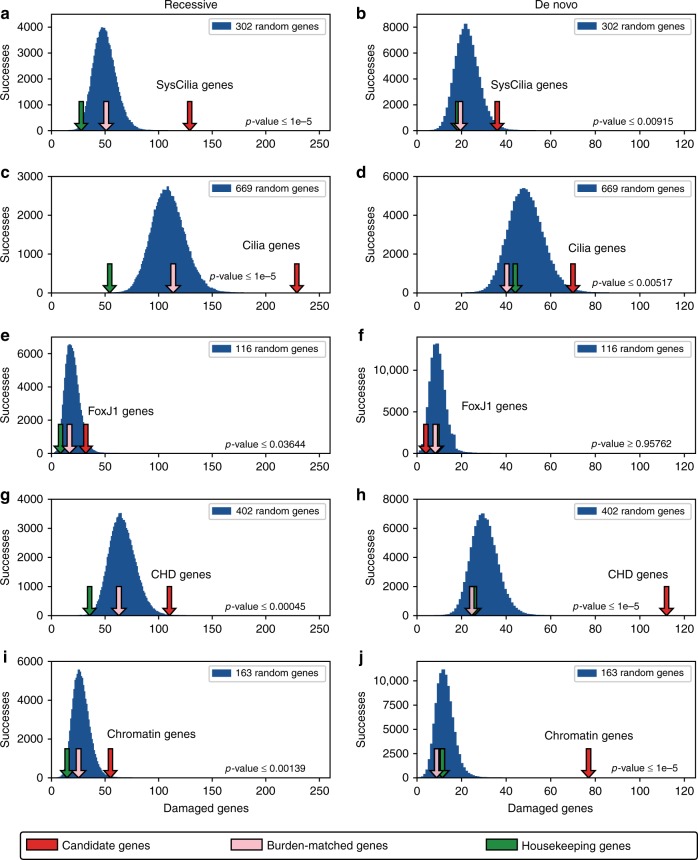
Enrichment profiles for damaged cilia and chromatin genes in CHD probands. Five candidate gene lists, SysCilia (302), Cilia (669), FoxJ1 (116), CHD (402), and chromatin-modifying (163) genes were tested for enrichment in damaging genotypes using 2391 congenital heart disease trios. The number of damaged genes discovered in the 2391 probands for each candidate gene list (red arrows) is compared to the distribution of damaged genes found using random gene lists of equal size (blue distributions, 100,000 independent random gene lists per distribution). **a**, **c** SysCilia and Cilia genes are highly enriched for damaged recessively inherited genotypes. **b**, **d** SysCilia and Cilia genes show only modest enrichment in de novo mutations. **e**, **f** FoxJ1-responsive genes are also modestly enriched for recessive variation but not for de novo variation. **g**, **i** CHD and chromatin-modifying genes are only modestly enriched for damaging recessive genotypes. **h**, **j** In contrast to cilia genes, known CHD and chromatin-modifying genes are highly enriched for damaging de novo mutations. Burden-matched control genes (pink arrows) are not significantly enriched for any gene set. Housekeeping genes (green arrows) are depleted for damaging recessive variation and have a typical amount of damaging de novo variation. All *p* values are obtained by empirical permutation

To further control for potential biases related to differences in burden or other factors intrinsic to the gene lists, we devised an algorithm for estimating the statistical significance and extent of enrichment of mutations in a given gene class (see Methods). The essence of the test lies in comparing the observed number of damaging genotypes (de novo or recessive) identified for a particular gene list, and comparing it to the number of genes with damaging genotypes in a randomly selected gene list of equal size. An empirical permutation test is used to estimate *p* values. Results are shown in Fig. 3. In each panel, the distribution of gene-hits for the randomly selected gene lists is shown in blue. Note that, as expected, the variance of these distributions fluctuates across panels due to the differing sizes of the gene lists and their intrinsic burden. Two controls are included in each panel: a set of housekeeping genes matched to the number of genes in each gene list (green arrows) and a burden-matched random list of genes (pink arrows); see Methods for an explanation of how the burden-matched lists were compiled. The housekeeping list provides a reference point for low burden genes (see Fig. 2b), whereas the burden-matched control list provides a reference for the expected number of damaged genes for an arbitrary list of genes equal in size and matched for burden.

Inspection of Fig. 3 reveals consistent trends across the different gene sets and panels for all control gene lists. The first general trend is that the housekeeping genes (green arrows) are generally located near the mean of the de novo distributions (right-hand panels in Fig. 3) and are shifted to the left in the recessive distributions (left-hand panels). In other words, the frequency of damaging de novo variants in housekeeping genes is not significantly different from the frequency of de novo variants observed genome-wide. On the other hand, housekeeping genes are depleted for damaging recessive genotypes compared to the genome as a whole. A simple explanation for this trend is that probands with recessive, damaging genotypes in essential housekeeping genes are not likely to have CHD and would not have been included in the PCGC cohort. The second general trend to note is that the burden-matched control lists (pink arrows) are located near the mean in every panel, and never differ significantly from it, meaning that differences in burden across the different gene lists cannot explain the significance of the observations (red arrows) discussed in the following paragraphs.

### SysCilia genes

For structural cilia genes, VAAST identified 129 damaged genes with recessive or compound heterozygous inheritance in 126 probands (Supplementary Data 2). These genes include several dyneins (*DNAH1, DNAH5, DNAH6, DNAH11, DYNC2H1*), other components necessary for ciliary assembly and vesicle trafficking (*CEP290, NPHP3, IFT172, IFT140, PCM1*), myosins (*MYO15A, MYO3B*), tubulins (*TUBB3, TUBGCP6*), and actin-associated proteins (*SYNE2*). The rate of recovery of recessive genotypes is significantly higher compared to the empirical distribution of damaged genes found using an equal number of randomly sampled genes (permutation *p* value < 1e^−5^; Fig. 3a). Moreover, an equal number of randomly selected burden-matched genes and a list of housekeeping genes showed no enrichment compared to the random distribution. In contrast, VAAST identified 36 damaged genes with de novo variants in 36 probands in the SysCilia gene list (permutation *p* value < 0.0092; Fig. 3b).

### Cilia genes

VAAST identified 229 damaged genes in 213 probands (8.9% of analyzed probands) with recessive genotypes in the expanded cilia gene list (Fig. 3c). The larger cilia list identified an additional 64 distinct damaged cilia-related genes not found in the SysCilia gene list alone, and once again the signal was highly significant (permutation *p* value < 1e^−5^). These genes include additional dyneins (*DNAH3, DNAH7, DNAH12*), tubulin modifying proteins (*TTLL4, TTLL5, TTLL10*), laminins and related proteins (*LAMA5, SUN1*), and spectrins (*SPTBN5*). By comparison, de novo variants were only modestly enriched in the Cilia list: 70 damaged genes in 69 probands (permutation *p* value < 0.0052; Fig. 3d).

### FoxJ1-responsive genes

Also included in Fig. 3 are results for a list of 116 genes that show at least a twofold change in expression when FoxJ1 is over-expressed or depleted in a zebrafish model. The forkhead box transcription factor FoxJ1 is responsible for the formation of motile cilia during early development and the subsequent control of left-right asymmetry^21,22^. Genes that are regulated by the FoxJ1 transcription factor may contribute to CHD through alteration of cilia function during development. Consistent with the other cilia gene lists, there is a modest signal for recessive genotypes (permutation *p* value < 0.036; Fig. 3e). This recessive signal, however, was much lower in magnitude compared to the other cilia-related gene lists. Also, consistent with the trends observed for the larger SysCilia and Cilia lists, we observe no significant enrichment for de novo variants in the FoxJ1 list (permutation *p* value > 0.958; Fig. 3f). Considering all cilia-related lists, 47 genes with damaging recessive genotypes occurred in multiple probands (Supplementary Data 3) and 14 probands had two or three damaged cilia genes (Supplementary Table 1).

### CHD genes

The CHD candidate gene list provides an opportunity to assess the relative contribution of de novo and recessive genotypes to a wider set of genes previously implicated in CHD^4,5,14–16^. This gene list includes 35 chromatin-remodeling genes, but does not include any SysCilia or Cilia-related genes (see Fig. 2a). Figure 3g and h thus provide a reference point that reflects the current, general consensus for candidate genes (apart from chromatin-remodeling genes alone) implicated in CHD. Damaging recessive genotypes are modestly, but significantly enriched, and damaging de novo genotypes are highly enriched.

### Chromatin-modification genes

Reciprocal trends with regard to the enrichment of de novo vs. recessive genotypes are observed for chromatin-modifying genes related to CHD. VAAST identified 55 genes with damaging recessive genotypes in 55 probands for chromatin-related genes, a modest but significant enrichment compared to the expectation (permutation *p* value < 0.0014; Fig. 3i). Consistent with prior publications that have documented an excess of de novo variants in chromatin genes^3–5^, the enrichment of de novo variants in the chromatin list exceeds that observed for recessive variants, with VAAST identifying 77 damaged genes in 76 probands (3.2% of all probands) having de novo damaging variants (permutation *p* value < 1e^−5^; Fig. 3j).

### Other gene pathways

**Fig. 4:**
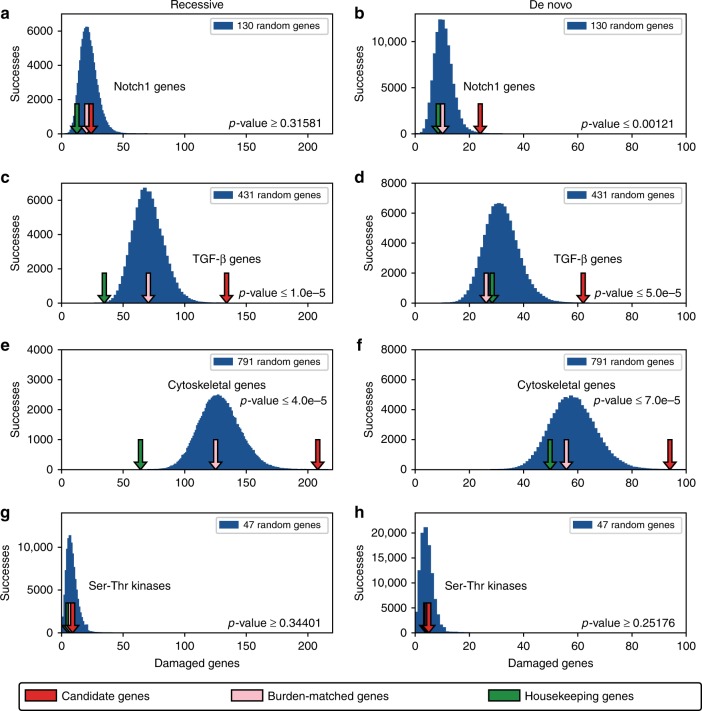
Enrichment profiles for additional genes and gene pathways. Several additional pathways implicated in congenital heart disease were tested for enrichment in damaging recessive genotypes and de novo mutations. **a**, **b** The Notch signaling pathway is enriched for de novo mutations (*p* value < 0.0012) but is not enriched for damaging recessive genotypes. **c**–**f** Genes involved in TGF-β signaling and non-ciliary cytoskeletal genes are moderately enriched in damaging recessive and de novo genotypes. **g**, **h** In contrast, receptor serine-threonine kinases show no enrichment for either damaging de novo or recessive genotypes. All *p* values are obtained by emperical permutation

Because the potential number of functional classes, pathways and expression categories is very large, we restricted our analyses to key categories of high a priori interest to CHD researchers. In addition to trends described above for cilia and chromatin-related genes, we also observed enrichment signals in Notch signaling pathway genes for de novo variants (permutation *p* value < 0.0012), for both recessive and de novo genotypes in TGF-β signaling genes (permutation *p* values < 1e^−5^ and <5.0e^−5^, respectively), and for non-ciliary cytoskeletal genes but not receptor serine-threonine kinases (Fig. 4a–h, Supplementary Data 4). The magnitudes of these signals, however, are smaller, and there is less difference between the inheritance modes of the enrichment signals as compared to cilia and chromatin-related genes.

### Additional gene lists

No enrichment of damaging recessive or de novo genotypes was found using the most highly expressed 400 genes from each of three different mature organs: left ventricle, brain, or liver. Embryonic high heart expression (HHE) genes were, as previously reported^4,5^, highly enriched for damaging de novo mutations (permutation *p* value < 1e^−5^). There was a modest enrichment of damaging genotypes in 86 candidate genes for autism spectrum disorder, recapitulating previous findings that showed damaging de novo mutations in chromatin genes that were common to patients with CHD and to patients with autism spectrum disorder^3^ (Supplementary Fig. 4, Supplementary Note 4). Moreover, there was no enrichment in damaging recessive genotypes and only modest enrichment in de novo mutations for the fibroblast growth factor (FGF), platelet derived growth factor (PDGF), and WNT signaling pathways (Supplementary Fig. 5, Supplementary Note 5).

### Relative contributions to CHD

The results shown in Figs. 3 and 4 naturally raise questions as to the relative contributions of recessive versus de novo genotypes to CHD. Within the SysCilia list, the rate of discovery of damaged recessive genotypes are enriched 1.57-fold compared to de novo mutations, whereas for the more inclusive Cilia-related list, the enrichment ratio decreases to 1.43-fold (averaging both lists gives a value of 1.50-fold). By comparison, de novo genotypes are enriched 3.19-fold in the chromatin gene list compared to recessive genotypes. Thus, the relative enrichment of damaging recessive genotypes within the cilia and cilia-related genes is about half (0.47) that of de novo variants within chromatin genes.

**Fig. 5:**
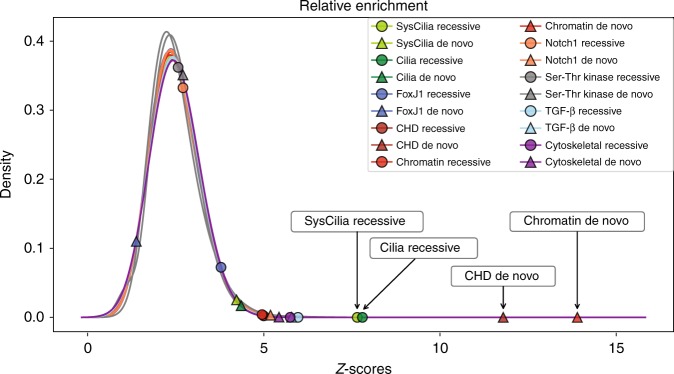
Relative enrichment of damaging recessive and de novo variation in CHD patients. To compare the findings among the distributions shown in Figs. 3 and 4, each distribution was normalized by *Z*-score transformation. Recessively inherited damaged genotypes found in SysCilia and Cilia genes are highly enriched in CHD probands (+7.7 SDs, *p* value < 1e-5). These genes show only marginal enrichment for de novo mutations as compared to the enrichment seen for damaging recessive genotypes. In contrast, there is strong enrichment for de novo mutations in chromatin-modifying and known CHD genes (+14.0 and +17.1 *SDs*, respectively, *p* value < 1e-5) but relatively moderate enrichment for damaging recessive genotypes. Other gene lists representing the Notch pathway,  the TGF-β signaling pathway, and cytoskeletal genes are also moderately enriched (+4.0 to +5.2 SDs), suggesting an important but more limited contribution to CHD as compared to cilia and chromatin genes. *P* values are obtained by permutation of the transformed distributions

These simple calculations, however, do not take into account differences in burden and size between gene lists. This can be accomplished by standardizing the distributions shown in Figs. 3 and 4 using *Z*-scores^23^, as shown in Table 1. The normalized distributions are shown in Fig. 5. Once normalized, the SysCilia and Cilia gene lists produce similar *Z*-score values of 7.73 and 7.95, respectively, for recessive genotypes. For the Chromatin gene list, the value is 17.10 for de novo genotypes (see Supplementary Data 5 for all *Z*-scores). The *Z*-score-derived enrichment ratio for the averaged Cilia recessive gene lists (7.84) to Chromatin de novo genotypes is 0.46*x*. This value is very similar to the non-burden adjusted value obtained using the VAAST genotype counts directly. That both approaches to the calculation give comparable values indicates that our analytical methods adequately controlled for differences in burden between the gene sets.

**Table 1:** Relative enrichment of damaging genotypes by gene list

Gene list	*Z*-score (recessive)	*Z*-score (de novo)	*p* value (recessive)	*p *value (de novo)
SysCilia	7.73	2.60	<1e-5	0.0094
Cilia	7.95	2.79	<1e-5	0.0103
FoxJ1	1.93	−1.67	0.0728	0.0273
CHD	3.75	13.95	0.0009	<1e-5
Chromatin	3.68	17.10	0.0027	<1e-5

*p* values obtained by Z-distribution permutation

Although the Z-scores provide means to compare relative enrichment of gene lists and gene pathways, they should not be interpreted as measurements of PAR. Considering genotypes found in all probands, the relative PAR estimates for damaging recessive and de novo genotypes are 8.9% and 3.2% for the Cilia and Chromatin-related gene lists, respectively. When considering PAR for categorical associations, the size of the gene list should be taken into account. For example, normalizing these estimates by the size of the cilia- and chromatin-related gene lists (669 vs. 163) produces similar per gene PAR estimates of 0.013 and 0.020 percent gene^−1^, respectively.

### Laterality defects and recessive genotypes

Previous studies have linked human ciliopathies to heterotaxy, and several studies have characterized CHD in a subset of those individuals^11,24,25^. Very recently, WES analysis of unrelated laterality patients with CHD identified inherited variation, including low-frequency recessive and compound heterozygous genotypes, as likely candidates for the disease^7^. To test the hypothesis that rare recessive and damaging genotypes in cilia genes are preferentially associated with laterality defects, we examined the association between having a laterality/heterotaxy phenotype and a damaged recessive genotype by gene list (Table 2).

**Table 2:** Association of CHD probands and laterality defects by gene list

Gene list	Number of genes	*p* value
SysCilia	302	0.0158*
Cilia	669	0.0377
FoxJ1	116	0.0719
CHD	402	0.2175
Chromatin	163	0.5009
Housekeeping	669	0.7233

Fisher’s test; *Significant with Benjamini–Hochberg correction, FDR = 0.1

We find that probands with damaged structural cilia genes are weakly associated with laterality defects. Probands with damaging recessive genotypes in the SysCilia genes are marginally more likely to have laterality/heterotaxy phenotypes than other phenotypes (*p* value < 0.016, Fisher’s test). It is essential to note that damaged cilia genes are found in probands of all phenotypic classes. Indeed, 3.9% of probands with conotruncal defects harbor a damaged cilia gene, compared to 1.6% of probands with a heterotaxy/laterality phenotype. Overall, though, laterality accounts for a much smaller fraction of all CHD than other phenotypes. The observed enrichment of cilia genes in laterality is driven by the relative proportions of probands harboring a damaged recessive cilia genotype. Collectively, these results suggest that the excess of laterality/heterotaxy defects associated with cilia genes is a consequence of the fact that dominant and recessive CHD have distinct functional signatures, a point we discuss in more detail below.

### Belief network-based analyses

We used a Belief network^26^ to further investigate the intertwined relationships between genotypes, gene functions, and phenotypes in our data. This Bayesian approach provides a means to tease apart the confounding effects of overlapping gene lists, and potentially confounding variables such as proband ancestry and exome capture methodology. Belief nets also avoid the well-known pitfalls associated with maximum likelihood estimates derived from limited numbers of observations, and provide a best-practice methodology for dealing with missing data^26,27^.

**Fig. 6:**
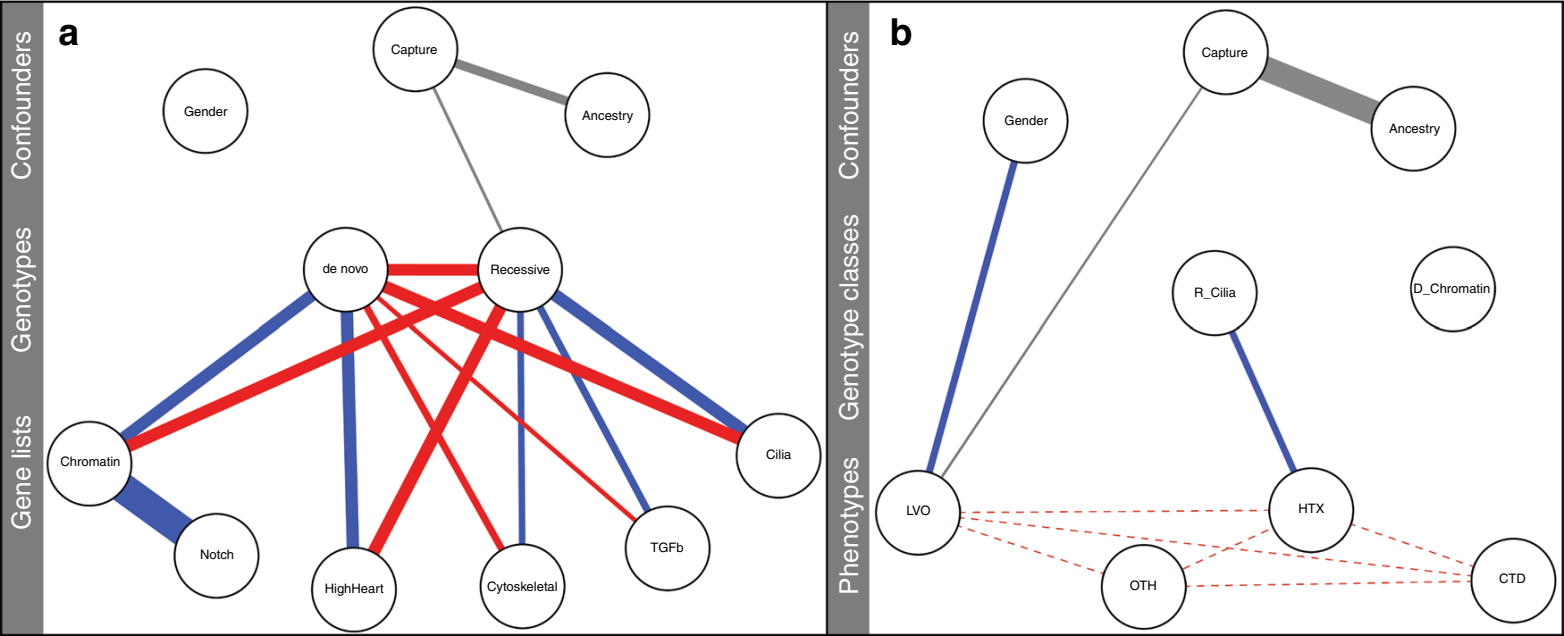
Genetic and phenotypic landscapes for 2391 patients with congenital heart defects nodes in these two Bayesian networks correspond to genotypes, gene functions, tissue of expression, phenotypes, and potentially confounding variables such as ancestry and gender. Connections, or edges, between nodes denote conditional dependencies. The width of an edge is proportional to the relative strength of the dependency, calculated as the average pairwise fold change in risk. Blue denotes positive dependencies; red denotes negative dependencies. Relationships between multistate nodes (gender, ancestry and capture method) are shown in gray, because the nature of an association (positive or negative) can vary by state, e.g. male or female for gender. Unconnected nodes indicate conditional independence. See text for additional details. **a** Genotypes and gene functions. Relationships between recessive and de novo damaging genotypes, and gene lists. Cilia: SysCilia and cilia-related genes; HighHeart: genes highly expressed in the embryonic heart; TGF-βː TGF-β pathway associated genes; Notch: Notch-pathway associated genes; Chromatin: chromatin-modifying and related genes; Cytoskeletal: cytoskeleton-related genes. **b** Genotype classes and phenotypes. Relationships between proband phenotypes, and recessive and de novo damaging genotypes in cilia and chromatin-related genes, respectively. HTX: heterotaxy; CTD: conotruncal defects; LVO: left ventricular outflow defects; OTH: other phenotypes. Dotted lines denote mutually exclusive categories

Figure 6a shows a best-fit belief net summarizing the relationships between proband genotypes and gene lists with significant contributions to CHD (see Fig. 5 for relative contributions). Also shown are potentially confounding variables such as gender, ancestry, and the capture method used for WES. As would be expected from Fig. 5, positive relationships are observed between de novo genotypes and chromatin-related genes, and reciprocally, recessive genotypes and cilia-related genes. Belief nets not only illustrate data trends, they also provide means to quantify them. For example, knowing that a proband has a damaging de novo genotype increases the probability that the proband has a damaged chromatin-related gene by about 50% compared the null expectation (*P*(chromatin |de novo)/(chromatin) = 1.48). Reciprocally, knowing that a proband has a damaging recessive genotype increases the probability that the proband has a damaged cilia-related gene by about 30% compared the null expectation (*P*(cilia|recessive)/*P*(cilia) = 1.3).

The network shown in Fig. 6a makes it clear that stratification between gene function and genotype is due to both positive and negative associations. Thus, knowing that a proband has a damaging de novo genotype, but not a recessive one too, increases the probability that the de novo genotype will lie in a chromatin-related gene from 1.48- to 2.6-fold, compared to the expectation given no knowledge of genotype, e.g. *P*(chromatin|de novo, ¬ recessive)/P(chromatin) = 2.6. Reciprocally, knowing that a proband has a damaging recessive genotype, but not a de novo also, increases the probability that the recessive genotype will lie in a cilia-related gene from 1.3-fold to 1.7-fold, compared to the expectation given no knowledge of genotype (*P*(cilia|recessive, ¬ de novo)/*P*(cilia) = 1.7).

The impacts of confounding variables, such as gender, sequencing methodology and ancestry, on these trends can also be explicitly addressed and controlled for by using a belief net. Figure 6a indicates strong stratification between sequencing capture and ancestry. For example, there is a twofold bias in the dataset as regards use of the xGEN IDT capture technology for probands of African ancestry, i.e., *P*(ancestry = African|capture = xGEN IDT)/*P*(ancestry = African) = 2.0. Importantly, specifying ancestry explicitly allows us to investigate its potentially confounding role on other trends. For example, knowing that a proband has African ancestry and has a damaging recessive genotype, increases the probability that the proband has a damaged cilia-related gene by 30% compared the null expectation. In fact, the same 30% increased probability holds true when specifying Asian or European ancestry (*P*(cilia|recessive, ancestry = African)/*P*(cilia) = *P*(cilia|recessive, ancestry = Asian)/*P*(cilia) = *P*(cilia|recessive, ancestry = Western European)/*P*(cilia) = 1.3). Thus, ancestry is not driving the association between cilia genes and recessive genotypes, nor is sequencing capture, as specifying ancestry explicitly negates any confounding effects of sequence capture on ancestry. Thus, the belief net informs us that while a disproportionate number of African-ancestry probands were sequenced using a unique capture method, neither ancestry nor capture method fundamentally impact the recessive cilia and de novo chromatin associations.

There also exists a strong positive conditional dependency between the Chromatin-related and Notch-pathway gene lists. The belief net provides the context to further understand these dependencies. For instance, if we know that a proband does not have damaging de novo chromatin variant, the probability of harboring a damaging Notch variant is 0.15 × (*P*(Notch|¬Chromatin, de novo)/*P*(Notch|Chromatin, de novo) = 0.15). The fact that this ratio is <1 indicates that the association between de novo genotypes and Notch-pathway genes is driven by de novo genotypes in genes shared in common between the chromatin and Notch gene lists (e.g. *CREBBP*, *HDAC2*, *HDAC7*, *KAT2A*, *TP53*).

We also observe that co-occurrence of damaging de novo and recessive genotypes within the same proband can complicate relationships between genotypes and gene functions. In Fig. 6a, for example, it appears the cytoskeletal genes are depleted, rather than enriched for de novo genotypes, in apparent contradiction to Fig. 4. Globally speaking this is true, as the probability of a damaging de novo cytoskeleton gene (without knowledge of a recessive variant) is 0.79 × (P(cytoskeleton|de novo)/*P*(cytoskeleton) = 0.79). However, restricting the calculation to probands with only de novo genotypes, knowing that a proband has a damaging de novo, but not recessive variant, increases the probability that the patient harbors a damaging de novo cytoskeleton variant to 1.3×, compared to the null expectation (*P*(cytoskeleton|de novo, ¬ recessive)/*P*(cytoskeleton) = 1.3). It is worth noting that double-hit individuals occur less often in the CHD cohort than would be expected by chance, by a factor of 0.73 × (*P*(recessive, de novo)/[*P*(recessive)*P(de novo)] = 0.73, *p* < 7.9e^−16^), suggesting that these individuals are being systematically excluded, perhaps because they tend to die in utero. Moreover, we also observe no increased probability of double-hit individuals to manifest an HTX phenotype, as *P*(HTX|R_Cilia, D_Chromatin)/*P*(HTX|R_Cilia) = 1.0. We cannot, however, fully exclude an important multi-genic contribution to CHD, because we did not assess the role of inherited (incompletely penetrant) dominant genetic variants.

As regards expression, de novo genotypes are about twice as likely to occur in embryonic HHE genes than are recessive ones. This trend is slightly more pronounced when gene function is taken into account; for example, *P*(HighHeart|chromatin, de novo, ¬ recessive)/*P*(HighHeart|cilia, recessive, ¬ de novo) = 2.8, in general agreement with previous reports^3–5^.

Finally, we asked how well the model in Fig. 6a explains the CHD dataset globally. The likelihood ratio test (LRT) is traditionally used to compare the relative fit of different probabilistic models to the data^27^. Belief nets provide an intuitive way to think about the capability of different models to describe the data, as alternative (nested) models can be produced simply by deleting some combination of edges from the net. For example, the LRT *p* value for the model presented in Fig. 6a is 3.9e^−91^ compared to one without any connections between the genotype nodes and the rest of net, meaning that genotype is a key feature of the CHD landscape. Inclusion of cilia genes also significantly increases the fit of the model compared to one without (LRT *p* value: 2.3e^−12^), as does adding the high-heart expressing, Notch, TGF-β, and cytoskeletal gene lists (LRT *p* value 4.8e^−39^).

Figure 6b shows a belief net summarizing the relationships between phenotypes, recessive genotypes in cilia genes (R_Cilia), and de novo genotypes in chromatin-related genes (D_Chromatin), together with potentially confounding variables such as ancestry, sequence capture methodology, and gender. The belief net highlights a positive association between heterotaxy and recessive cilia genotypes. This same association is detectable via traditional hypothesis testing (Table 2), but the belief net provides means to quantify relative risk. For example, having a recessive cilia genotype increases the relative risk of heterotaxy 1.4-fold compared to a de novo mutation in a chromatin-related gene, (*P*(HTX|R_Cilia)/*P*(HTX|D_Chromatin)). Risk estimates are not absolute, but dependent upon the dataset used to estimate them. In light of this fact, the 1.4-fold increase in LVO phenotypes observed for males (e.g., *P*(LVO|Gender = male)/*P*(LVO|Gender = female) = 1.4) can be used to provide context for the strength of the HTX-cilia-recessive association. The gender bias for LVO phenotypes is well described, with a 2–4-fold male predilection, depending upon the LVO lesion^28–30^. Thus, the association between HTX and cilia-related recessive genotypes is similar in magnitude to that observed between LVO phenotypes and gender in this cohort.

## Discussion

We carried out a gene-burden aware quantitative analysis of 2391 CHD trios to discover and quantify relationships between gene functions, genotypes, and CHD phenotypes. We find that, as a class, cilia and cilia-related genes are enriched for rare, damaging recessive variants. This increased burden has interesting ramifications for human CHD, suggesting that cilia and cilia-related genes may provide a reservoir of rare and potentially damaging variation that is segregating in the population, with recessive homozygous and compound heterozygous combinations leading to CHD. Moreover, while enriched for damaging, rare recessive genotypes, cilia and cilia-related genes are less enriched for damaging de novo variants. Within the PCGC cohort, we found that 8.9% of probands harbored a damaging recessive genotype in cilia-related gene, while 3.2% harbored a damaging de novo variant in a chromatin-modifier. Correcting for the size of the cilia- and chromatin-related gene lists produces similar per gene PAR estimates (~0.013 and ~0.020 percent gene^−1^, respectively).

The overall low recurrence rate among CHD loci in the PCGC cohort^3–5^ suggests that more loci await discovery. Previous studies have implicated a variety of genes highly expressed in the developing heart as important candidate CHD disease-causing genes^15^. Our HHE and CHD gene lists contain many of these previously reported genes and like chromatin-modifying genes, damaging de novo variants in these genes are enriched in the PCGC cohort, with less enrichment observed in recessive genotypes. The genetic enrichment signals reported here are strongly weighted towards chromatin-modifying and cilia-related genes, with weaker, but significant, associations also detected in other gene classes relevant to CHD. The magnitude of genetic enrichment suggests that future de novo and recessive CHD-causing mutations will lie predominately in chromatin-modifying and cilia-related gene classes. That being said, the recent report^31^ of a variably expressed left ventricular non-compaction cardiomyopathy phenotype caused by heterozygous mutations in three separate genes (*MYH7*, *MKL2*, and *NKX2–5*) also emphasizes the need for methods that incorporate oligogenic and polygenic risk modeling. Moreover, future WGS approaches are likely to identify genomic loci not assessed in this analysis, such as promotors, enhancers and large structural variants.

While there is no single, overarching genotype–phenotype correlation throughout the dataset, we find that damaging recessive cilia genotypes in CHD probands show enrichment in laterality defect phenotypes, similar to recent reports^7^, although other CHD phenotypes also harbor damaged cilia genes. Thus, proband phenotypes reflect, in part, the distinct functional signatures of recessive and de novo CHD. Collectively, our findings show that amid the genetic and phenotypic heterogeneity of CHD there exists a network of highly significant associations between genotypes, gene functions, and phenotypes.

## Methods

### Participants

Patients were diagnosed, phenotyped, and recruited from PCGC centers and regional hospitals under institututional review board approved protocols from Boston’s Children’s Hospital, Brigham and Women’s Hospital, Children’s Hospital of Los Angeles, Children’s Hospital of Philadelphia, Columbia University Medical Center, Great Ormond Street Hospital, Icahn School of Medicine at Mount Sinai, Rochester School of Medicine and Dentistry, Steven and Alexandra Cohen Children’s Medical Center of New York, and Yale School of Medicine ([ClinicalTrials.gov] Accession number: NCT01196182)^3–5^. All patients or the patient’s parent(s) provided informed consent. CHD probands were classified into four major groups based on their cardiac phenotype: conotruncal disorders (CTD); left ventricular obstructive disorders (LVO), heterotaxy/laterality defects (HTX), or other (OTH, including atrial septal defect (ASD)). We defined heterotaxy/laterality as reported in Jin et. al. (2017) and included the following diagnoses: dextrocardia, situs inversus/ambiguous, atrial isomerism, asplenia/polyspenia, and all transpositions of the great arteries (TGA). The HTX diagnoses were often associated with AV canal defects, pulmonary atresia, or anomalous pulmonary venous drainage. HTX did not include isolated ASD.

### Variant calling

Raw sequencing data for all patients and parents were downloaded in fastq format from the PCGC HeartsMart database. Sequence data were aligned to the human genome reference sequence (hg19). Briefly, aligned BAM files underwent INDEL realignment and base recalibration. Each well-formed BAM file was processed with the Genome Analysis Tool Kit (GATK) haplotype caller to produce a sample GVCF file. The GVCF files were combined and jointly genotyped in two batches along with samples from the 1000 genome project (CEU and GBR). The GATK variant recalibrator was used to reduce potential false positives calls in the dataset. Tranche values were set to 99.5 and 99.0 for SNPs and INDELs, respectively. To reduce processing time, UGP pipeline steps were parallelized over hundreds of compute cores at the University of Utah Center for High Performance Computing [www.chpc.utah.edu]. Variants were normalized and decomposed using the vt program^32^. Annotation was applied to normalized variants with the Variant Effect Predictor (VEP) version 90 (ref. ^33^).

### Quality control

Quality control metrics were first applied to each of 2823 trios (see Supplementary Fig. 1, Supplementary Note 1). Relatedness among trio members was assessed as a kinship coefficient using the KING algorithm^34^. Trios with unrelated parent-offspring kinships estimates (<0.0884) were removed. These trios may represent families with adoptions, step-parents, sample-swaps, or incorrect paternity. Trios with parent–parent or parent–offspring with kinship estimates exceeding 0.35 were likely to be a result of a sample switch and were removed. Four trios with first cousin parents were retained. Trios in which the proband was diagnosed with Down syndrome, DiGeorge syndrome, or 22q micro-deletion/duplications were removed. Finally, trios with excessive exonic de novo mutations (>10) or very few recessive genotypes (<30) were removed. A final set of 2391 trios was used for analyses. Ninety percent of these trios (2157) have been included in previous reports^3–5^.

### Variant impact scoring

The 2391 trios were analyzed as trios using the VAAST Variant Prioritizer (VVP)^35^ and VAAST burden tests. A typical VVP command was: VVP -d vvp_v2_background/1KG.050417.vvp.db -i 1-00004.vcf.gz -v CSQ,3,6,0,15 -c -n 1 -o 1-00004.scored_variants.out>1-00004_VVP.out. All coding and splice-site SNVs and INDELs in each of the final 2391 probands over the entire exome were analyzed to assess the impact of each variant relative to variants seen in a background population of ~2500 samples (1000 genomes phase3). The output from VVP produces a VVP score of 0–100 (100 being the most damaging) for each variant under hemizygous, heterozygous, and homozygous inheritance models, accounting for the amino acid substitution, evolutionary conservation of the variant, and the frequency of the variant in the background population.

### Burden testing

Output from VVP was then used as input into the Variant Annotation Analysis and Search Tool (VAAST, version 3)^20,36^. VAAST is a gene-burden test^19^ that ranks the probability that a gene is damaged based on the genotypes found in the gene, the frequency of those genotypes in a background population, the amino acid substitution of each variant in that gene relative to variants found in the background samples, and the phastCon cross-species conservation at the variant site. VAAST assesses the genotypic burden in each gene relative to gene burden in the background population. This burden-based approach is especially relevant for assessing the impact of compound heterozygous genotypes because variant prioritization tools do not judge the impact of combinations of alleles. VAAST overcomes this pitfall by assaying each locus and its alleles in a combinatoric fashion and then permutes against a background population to identify combinations of alleles (burden) that may be disease causing (see ref. ^19^ for more on these points). A typical VAAST command was: VAAST -i 1-00004.scored_variants.out -d vvp_v2_background/1KG.050417.vvp.db -t 3 -b 2504 -n 1 -e r -f t -r 1 -m 2 -w 0 > 1-00004.burden.out 2 > 1-00004.burden.error. The output from VAAST produces a composite likelihood ratio score and a permutation-based *p* value for every gene taking into account an inheritance model and every possible combination of allelic variants in the proband relative to all possible combinations of allelic variants in background population, in this case, the ~2500 1K genomes samples.

### Integration of phenotype data with burden test results

The Phenotype Driven Variant Ontological Re-ranking Tool (PHEVOR) was used to re-rank genes from the VAAST output based on the posterior-probability that the gene was associated with the proband’s phenotype^37^. PHEVOR utilizes Human Phenotype Ontology (HPO) and Gene Ontology (GO) terms to prioritize genotype–phenotype associations. Phenotype-specific HPO information for each of the major cardiovascular phenotypes (CTD, LVO, HTX, and OTH) was used for re-ranking VAAST output. The HPO node ids used for the PHEVOR re-ranking were: Conotruncal Defects (CTD) [HP:0001669, HP:0001719, HP:0001660, HP:0001636, HP:0004414]; Left Ventricular Obstructive disorders (LVO) [HP:0001680, HP:0001647, HP:0001682, HP:0001706, HP:0004381]; Heterotaxy (HTX) [HP:0001669, HP:0001642, HP:0001643, HP:0011599, HP:0010772,HP:0012020, HP:0002101, HP:0011565, HP:0001696, HP:0010452, HP:0001674, HP:0001746, HP:0001629, HP:0004935, HP:0001748, HP:0001631, HP:0011537, HP:0011536]; Atrial Septal Defects (ASD) [HP:0001631, HP:0001684]; or other defects (OTH) [HP:0010316, HP:0001642, HP:0001674, HP:0006695, HP:0010772, HP:0011662, HP:0004935, HP:0001629]. The most damaged genes (up to 100) from the VAAST and PHEVOR analyses for every trio were retained and managed in a local MySQL database.

### Variant adjudication

To confirm the validity and inheritance of all recessive and de novo mutations identified by VAAST and PHEVOR, each variant from each gene was tested with GRAPHITE (D. Lee and G. Marth; [github.com/dillonl/graphite]). Graphite uses a local graph-based Smith–Waterman alignment to assess the sequencing reads at candidate variant position. Briefly, all sequencing reads in a 3 kb window around the variant were realigned in a variant graph using a Smith–Waterman algorithm. Only sequencing reads with a ≥95% Smith–Waterman alignment were used. Recessive variants with at least six supporting reads in each trio member that had at least two supporting reads on each strand and that demonstrated correct inheritance were retained. The de novo variants were required to have at least eight reads in the proband, and no reads in the parents. GRAPHITE adjudicated de novo variants were retained if also confirmed in the integrated genome viewer (IGV) or previously reported by the PCGC^3^. We tested 12 graphite-verified recessively inherited variants in three CHD genes (*DNAH5*, *CHD8*, *KMT2D*) and eight de novo variants in chromatin-related genes by Sanger sequencing. One variant could not be amplified. Eighteen of the remaining 19 variants were confirmed, thus yielding a 95% confirmation rate following GRAPHITE adjudication and IGV validation (Supplementary Data 6).

### Criteria for identification of damaging genotypes

A VAAST *p* value of ≤0.005 was used to classify genes as damaged. This VAAST *p* value threshold was calibrated against a previously published independent assessment of damaged genes causing CHD in PCGC patients^3–5^. A PAR filter of ≥0.005 was used to filter genotypes to eliminate genes with excessive variation attributable primarily to incorrect read mapping. We excluded mucins, olfactory receptors, any recessive genotype where either allele could be considered a common polymorphism (maf ≥ 0.05). Note that de novo and recessive genotypes are scored using the identical process and parameters, meaning that recessive and de novo genotype scores and *p* values are directly comparable. A total of 3083 recessive and 1351 de novo genotypes were identified and retained for enrichment-by-gene-list analyses. An estimated PAR of 3.2% for damaging de novo variants in chromatin-related genes is consistent with the PCGC’s previously reported estimate of 2.3%, which screened a smaller, less inclusive candidate list of chromatin-related genes using a different definition of deleterious missense variants^3–5^. In contrast, we identified many more candidate recessive genotypes on average, 1.3 proband^−1^, compared to 0.2 proband^−1^ reported previously^3^. The increased numbers result from VAAST’s ability to identity compound heterozygous genotypes for which the combined burden of two moderately damaging variants is equal to that of a single, severely damaging de novo variant^19,20,35,36^. By contrast, the PCGC’s previously reported recessive genotypes^3^ were restricted solely to cases for which both variants were loss-of-function alleles or missense variants predicted to be maximally damaging by MetaSVM.

### Population genotype frequencies

The recessively inherited damaging genotypes identified here comprise allelic combinations that are very rare in the general population. These genotypes are predominantly compound heterozygotes (88%) in the CHD patients. Eighteen percent of all identified alleles are not found in the gnomAD database. The variant effect predictor (VEP) annotates 13% of the alleles as high impact, 87% as moderate impact, and 0% as low impact variants. Most alleles (83%) have no reported homozygotes. After assigning a conservative allele frequency of 1/100,000 to alleles not found in the gnomAD database, we calculated a median expected genotype frequency for simple recessive and compound heterozygous genotypes of 8.7e-6 and 1.1e-7, respectively. Thus, most reported genotypes will be found in fewer than 1 in 100,000 individuals in the general population (Supplementary Fig. 6, Supplementary Note 6). Additionally, discovery of these damaging genotypes was not biased toward any particular population (e.g. populations with admixture or high heterozygosity) but was proportional to the ethnic distribution of the PCGG cohort as a whole (Supplementary Fig. 7, Supplementary Note 7, and Supplementary Data 7).

### Candidate gene lists

Several gene lists were utilized to enhance the search for genes involved in CHD (see Supplementary Data 1). A list of 302 published and well-characterized structural cilia genes (SysCil 2.0) was assembled from the literature^13^. An additional 367 cilia and potential cilia genes were identified by a GO-ontology search for cilia genes in model organisms (zebrafish and mouse). These additional genes include various gene classes such as dyneins, tubulin ligases, spindle proteins, regulatory proteins, and others, linked to cilia through the HPO and GO ontologies and model organisms. Orthoretriever was used to convert model organism genes to human genes. Several genes with more than three orthologs in either conversion direction were omitted. Recently, 61 mouse cilia genes associated with CHD were identified^11^. These additional cilia-related genes found in zebrafish and mouse models were added to the 302 SysCilia genes to create an expanded list of 669 cilia and cilia-related genes. A list of genes that were two-fold up- or down-regulated in response to forkhead box transcription factor FoxJ1a over-expression and knockdown in zebrafish was compiled from RNA-Seq and RNA-tiling expression results^38^. A list of 402 genes associated with CHD in humans or other organisms was compiled from published sources^4,5,14–16^. The CHD gene list contains a variety of genes encoding structural molecules, signaling molecules, and transcription factors, but does not overlap with the cilia genes defined in our cilia lists. A chromatin gene list included 163 chromatin-modifying genes defined by the PCGC^3–5^. Genes in this list include histone acetylases, histone deacetylases, SWI/SFN chromatin packaging complex proteins, actin methyltransferases, DNA-helicases, 16 transcription factors, etc., related to the modification of chromatin. The Notch gene list was hand curated. The cytoskeletal, Ser-Thr kinases, and TGF-β genes were assembled using the [reactome.org] and [genenames.org] pathway databases. Cancer-related genes were excluded. Highly expressed genes in the mature heart, liver, and brain were identified using the Genotype-Tissue Expression (GTEx) database [gtexportal.org/home/datasets]. High-ranking autism candidate genes (categories 1 and 2) were identified and assembled by the Simons Foundation Autism Research Initiative (SFARI, [https://www.sfari.org/resource/sfari-gene/]).

### Control gene lists

To create control gene lists, human housekeeping genes that show consistent expression over different cell types and conditions were randomly selected from published housekeeping gene lists^18^. To construct random gene lists with an equal genetic burden to the SysCilia, Cilia, FoxJ1, CHD, Chromatin, and other tested gene lists, rare variants (maf ≤ 0.005) found in the ExAC/gnomAD database^39^ were summed over the largest coding transcript of every gene. This sum was then divided by transcript length to produce a burden ratio for each gene. The mean burden ratio and corresponding standard deviation for each decile of the distribution of each gene list was matched by randomly sampling genes from the whole genome (excluding genes in the test list). By using a sampling approach, a list of random genes equal in size and matched for burden was created for each gene list tested for enrichment.

### Permutation testing

The permutation analyses were performed using a random sampling/permutation strategy. A gene list containing 18,876 RefSeq genes was first created. For each experimental gene list (SysCilia, Cilia, FoxJ1, Chromatin, etc.) of size *N*, random samples of equal size were drawn from the 18,876 genes. The database of all recessive or de novo variation was queried with the random gene lists to identify damaging recessive and de novo variation (VAAST *p* value ≤ 0.005). This process was repeated 100,000 times, each time with an independently generated random gene list, to create an empirical distribution of the number of damaged genes (VAAST *p* value ≤ 0.005) for a gene list of size *N*. Genes with a PAR of ≥0.005 were excluded. To test whether the PCGC probands show enrichment in the SysCilia, Cilia, FoxJ1, CHD, Chromatin, and other gene lists, the actual number of damaged genes (VAAST *p* value ≤ 0.005) found for each list was compared to the distribution of damaged genes found using random genes. An empirical *p* value was calculated as *p* value = (*d* + 1)/(*P* + 1), where *d* is the number of damaged random genes that exceeded the actual number of damaged genes in the test list, and *P* is the total number of random permutations. The empirical null distributions are good fits to Gaussian distributions. Thus, each distribution can be standardized by subtracting the mean and dividing by the standard deviation, i.e., *Z*-scores, allowing us to rank and order of *p* values of different tests.

### Belief nets

We learned the structure of the Bayesian networks using the package “bnstruct”^40^, which provides an exact search and an Akaike Information Criterion (AIC)-based scoring function (Silander-Myllymaki algorithm). The exact search algorithm explores the entire space of conditional dependencies in order to discover the optimal network structure for the data. Parameter learning for this optimal network is accomplished using the junction tree algorithm^26^ provided by gRain package^41^. We use the same package for our inference and risk calculations. To obtain the edge frequencies of the learned structure, we used bootstrap estimates provided by the “bnlearn” package^42^. In this setting we performed 10,000 bootstrap replications. Bootstrapped structures were calculated with a heuristic hill climbing search algorithm that uses AIC for optimization. Belief networks by definition represent conditional dependencies in the dataset as a directed acyclic graph (DAG); however, it is important not to confuse directionality with causality or temporal ordering. In keeping with best practice, the belief networks are visualized in their undirected, moralized form, in which every node is connected to its Markov blanket.

### URLs

Software used for the analyses may be obtained from the following sites: GATK: [software.broadinstitute.org/gatk/download/], VEP: [uswest.ensembl.org/info/docs/tools/vep/script/vep_download.html], VVP and VAAST: [github.com/Yandell-Lab/VVP-pub], PHEVOR: [www.yandell-lab.org/software/phevor.html], GRAPHITE: [github.com/dillonl/graphite], MySQL: [dev.mysql.com/downloads/repo/yum], R: [www.r-project.org] (bnlearn, gRain, and bnstruct libraries), Julia: [julialang.org].

### Reporting summary

Further information on research design is available in the Nature Research Reporting Summary linked to this article.

## Supplementary information

Supplementary Information

Supplementary Dataset 1

Supplementary Dataset 2

Supplementary Dataset 3

Supplementary Dataset 4

Supplementary Dataset 5

Supplementary Dataset 6

Supplementary Dataset 7

Reporting Summary

Description of Additional Supplementary Files

## Data Availability

The sequencing data used in this analysis may be downloaded, with committee-approved access, from the database of Genotypes and Phenotypes (dbGaP) [www.ncbi.nlm.nih.gov/] (accession numbers phs000571.v5.p2). Additional data files may be obtained from the authors upon request.
