# Supplementary material for: De novo and recessive forms of congenital heart disease have distinct genetic and phenotypic landscapes
Source: Nat Commun. 2019 Oct 17;10:4722. doi: 10.1038/s41467-019-12582-y (PMC6797711; doi:10.1038/s41467-019-12582-y)
Supplement: Supplementary file 1 — Supplementary Information [file 41467_2019_12582_MOESM1_ESM.pdf]

De novo and recessive forms of congenital heart disease have distinct genetic and phenotypic landscapes

Watkins et al.

Supplementary Information

Supplementary Note 1

Hypothesis testing for enrichment may be strongly influenced by the presence of data artifacts and outliers. For instance, an undeclared step-mother in a trio would be expected to lead to a greater than average number of *de novo* mutations detected in the offspring. We sought to minimize the influence of these factors by screening for kinship, the number of *de novo* mutations, and the number of recessive genotypes in each trio prior to performing any enrichment analysis. To reduce these potential sources of error, pairwise kinship coefficients were estimated for trio members using the KING algorithm[1]. Trios with parent-offspring kinship estimates < 0.0884 (less than 3rd-degree relatedness) were removed from the final analysis data set. Probands with 10 or more *de novo* mutations were also eliminated. Some trios showed a very low number (< 30) of recessively-inherited genotypes scored by VAAST and were removed. **Supplementary Figure 1** shows the distributions of these parameters in the final data set of 2,391 trios.

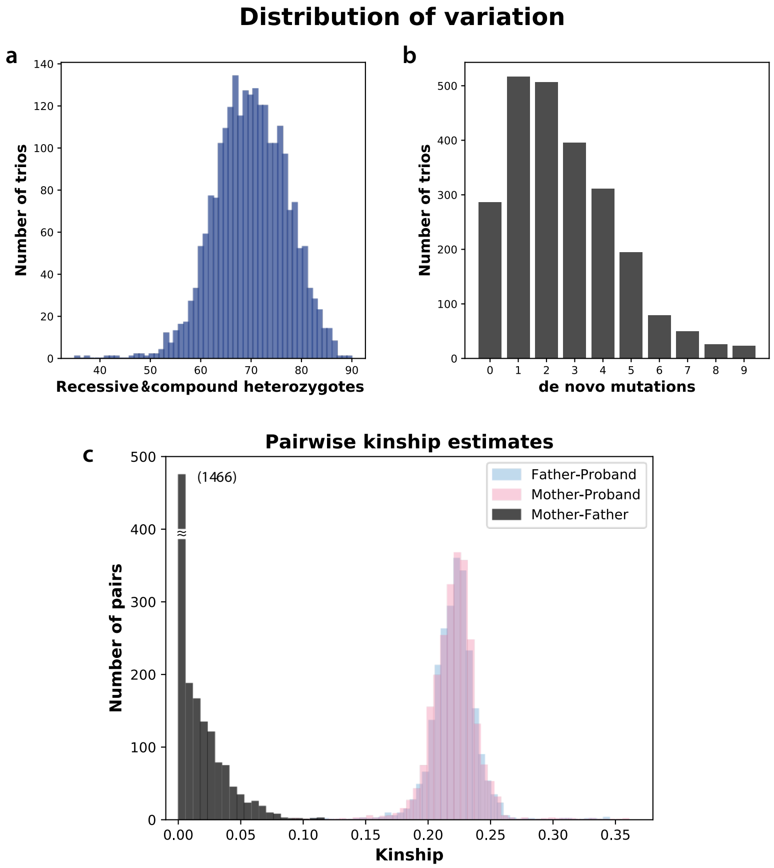

Supplementary Figure 1. a. The distribution of the number of recessive and compound heterozygotes scored by VAAST at any p-value. **b.** The distribution of all *de novo* mutations scored by VAAST at any p-value. **c.** The distributions of parent-parent (black), mother-proband (pink), and father-proband (blue) kinship coefficient estimates for the 2,391 trios used in the analysis.

Supplementary Note 2

The discovery of damaging variation in genes related to cilia function and structure may be influenced by the genetic burden for a given gene. This burden is often, but not always, positively correlated with the size of the gene. For example, some cilia-related genes have long coding regions which provide larger targets for new mutations (e.g. SYNE2 (~21 kb) and DNAH5 (~15.6 kb)). To better understand genetic burden in genes used for the enrichment analysis, the burden for each gene list was quantified using data from the gnomAD database (**Supplementary Figures 2**).

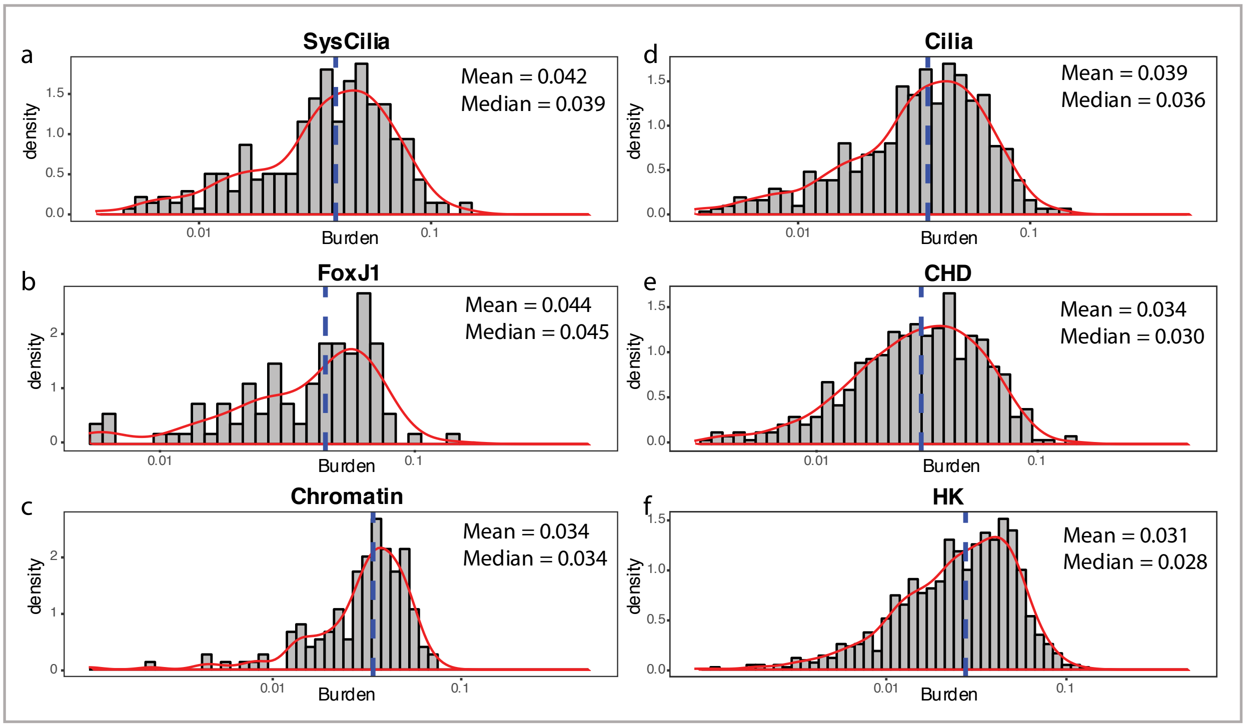

Supplementary Figure 2. The distribution of known genetic burden for rare alleles in each gene list. The relative genetic burden for key gene lists used in the enrichment analysis was quantified using variation from the gnomAD database. The number of genetic variants with an allele frequency of ≤ 0.005 were summed and divided by the length of the longest transcript for each gene. The distribution of this burden ratio is shown in log scale for each gene list. The dotted blue line indicates the mean. Kernel density estimation is shown by the red curves. **a, b, c, d, e, f**. The overall parameter estimates for the probability density function were similar among the lists even though the mean and median values are marginally higher for the SysCilia, Cilia, and FoxJ1 genes than for chromatin-modification, known CHD genes, or housekeeping genes. These results show that there is only a slight overall increase in genetic burden for ciliary genes as compared to other genes used in the analysis.

Supplementary Note 3

The VAAST algorithm is designed to control for differences in genetic burden among genes. The VAAST p-values should not be more significant simply because a gene or set of genes are larger or have a greater mutational load. To verify that genes identified by VAAST as having damaging genotypes (p-values ≤ 0.005) are not over-represented in certain gene lists containing large genes (e.g. cilia genes), VAAST p-values were analyzed as a function of genetic burden (normalized by transcript length) for six key gene lists (**Supplementary Figure 3**).

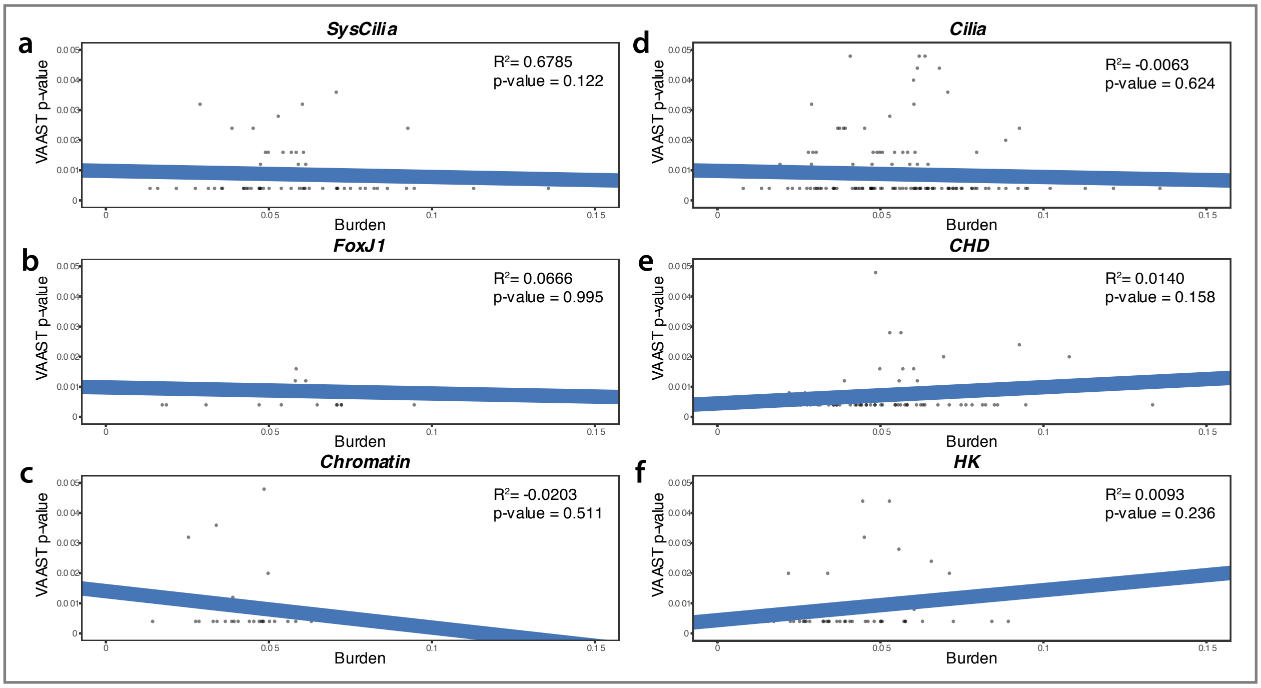

Supplementary Figure 3. VAAST p-value estimates vs gene burden. The VAAST p-value estimates for damaged genotypes is plotted as a function of gene burden by gene list. Burden is calculated as the number of rare variants (maf ≤ 0.005) found in the gnomAD database[2] for each gene divided by the length of the transcript for that gene. **a, b, c, d, e, f.** Least squares regression for each of the primary gene lists for the study is indicated by the blue lines. The regression lines show that there is no significant relationship between the amount of burden and the VAAST p-values for cilia-related gene lists or for other key gene lists (all p-values > 0.1, linear-model).

Supplementary Note 4

Our burden testing approach for enrichment is extensible to hypothesis testing for lists of genes from other gene pathways and experimental conditions (e.g., RNAseq, GWAS candidates, etc.) Testing of additional candidate gene lists may be useful to further define pathways involved in CHD or to exclude pathways and gene list candidates. This approach is well suited to complex disease where single gene approaches are explanatory for only a small fraction of cases. To further investigate additional sets of genes that, *a priori*, are expected to show little enrichment for damaged genes in CHD patients, we tested the top approximately two percent of expressed genes from mature brain, mature liver, and mature heart left ventricle. We also tested genes with high expression in mouse embryonic heart (HHE genes)[3] and a set of 86 genes that are associated with autism spectrum disorder [sfari.org/resource/sfari-gene/]. Previous work has shown a significant number of damaging *de novo* mutations in genes associated with autism in children with congenital heart disease[4]. **Supplementary Figure 4** demonstrates that genes expressed in mature brain, mature liver, and mature heart left ventricle are not enriched for damaging recessive or *de novo* genotypes in CHD patients. There is, however, significant enrichment in the HHE genes and the autism associated genes in CHD patients, confirming previous findings. (see **Supplementary Data 4** for all p-values).

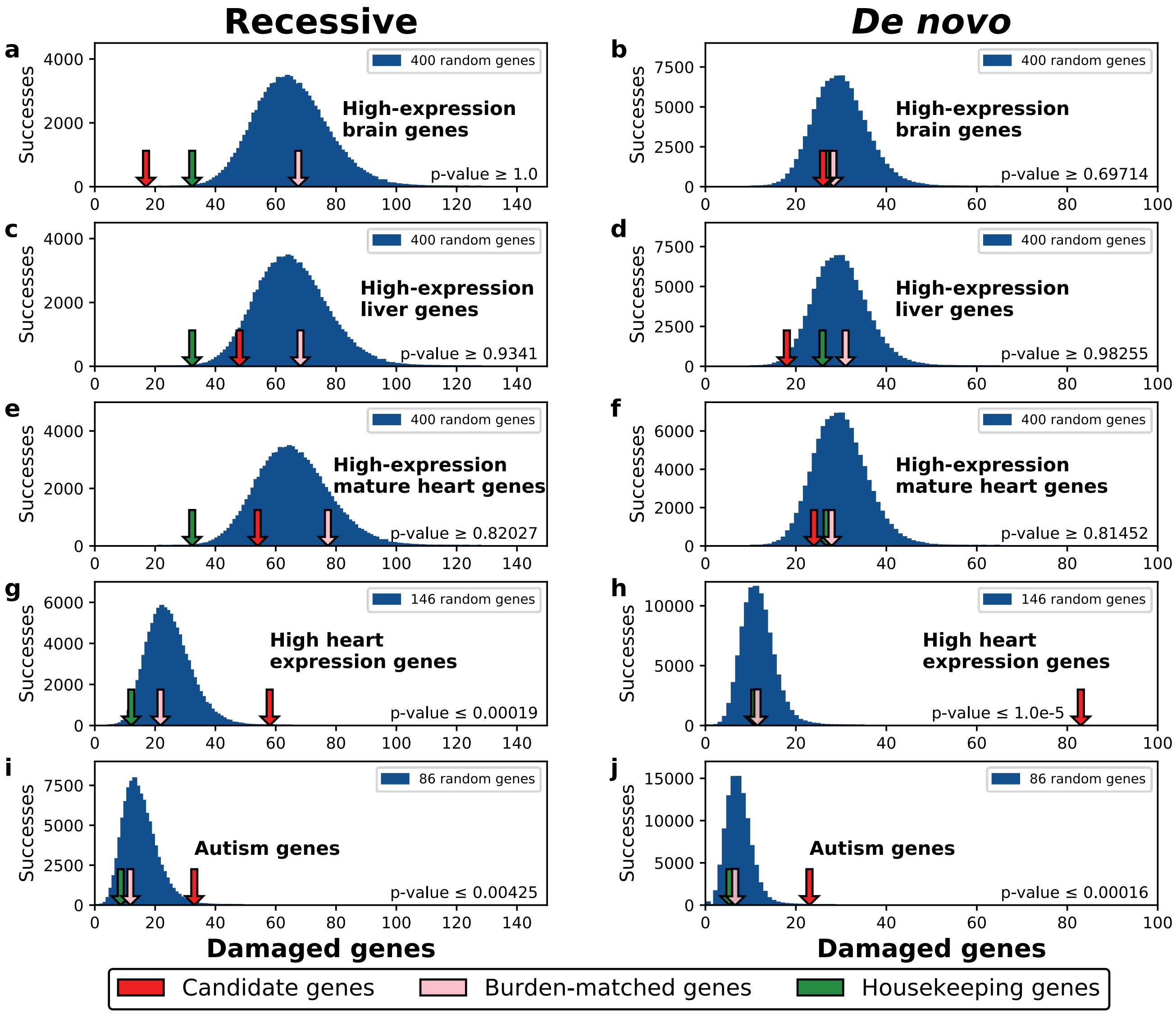

Supplementary Figure 4. Enrichment analysis in additional gene pathways and gene lists. a, c, e. The top approximately two percent of genes expressed in mature brain, mature liver, and mature heart left ventricle are not enriched for damaging recessive genotypes. **b, d, f.** Likewise, mature brain, mature liver, and mature heart left ventricle are not enriched in *de novo* mutations the in 2,391 CHD patients. **g, h.** Genes with high expression in the embryonic heart (HHE genes) are enriched in damaging recessive genotypes and are strongly enriched in damaging *de novo* genotypes. **i, j.** A list of 86 genes previously associated with autism spectrum disorder shows modest yet significant enrichment for damaging recessive and *de novo* genotypes.

Supplementary Note 5

The CHD genes presented in the main text contain genes involved in cellular signaling. Many of these signaling genes are components of classical signaling pathways and play key roles in normal development and cellular differentiation. To investigate the relative contribution of four classical signaling pathways to CHD, genes of the fibroblast growth factor (FGF), hedgehog (HH), platelet-derived growth factor (PDGF), and Wnt (WNT) signaling pathways were identified using the reactome [https://reactome.org/PathwayBrowser/] and PANTHER pathway tools [http://www.pantherdb.org]. Each signaling pathway gene list was tested for enrichment in damaging recessive and damaging *de novo* genotypes in the 2,391 CHD trios (**Supplementary Figure 5**). These signaling pathway genes showed no enrichment in damaging recessive genotypes. The PDGF and WNT pathways have a substantial depletion in the number of damaging recessive genotypes, similar to housekeeping genes. This result suggests that many genes of the WNT and PDGF pathways are essential to normal development and overall viability. The WNT, PDGF and FGF signaling pathway showed a modest but significant enrichment in damaging *de novo* mutations. The magnitude of the enrichment was similar to the Notch1 and TGF-B pathways. On average, approximately half the observed enrichment signal seen in the *de novo* analysis above can be accounted for by the 402 genes in our list of known CHD genes presented in the main text.

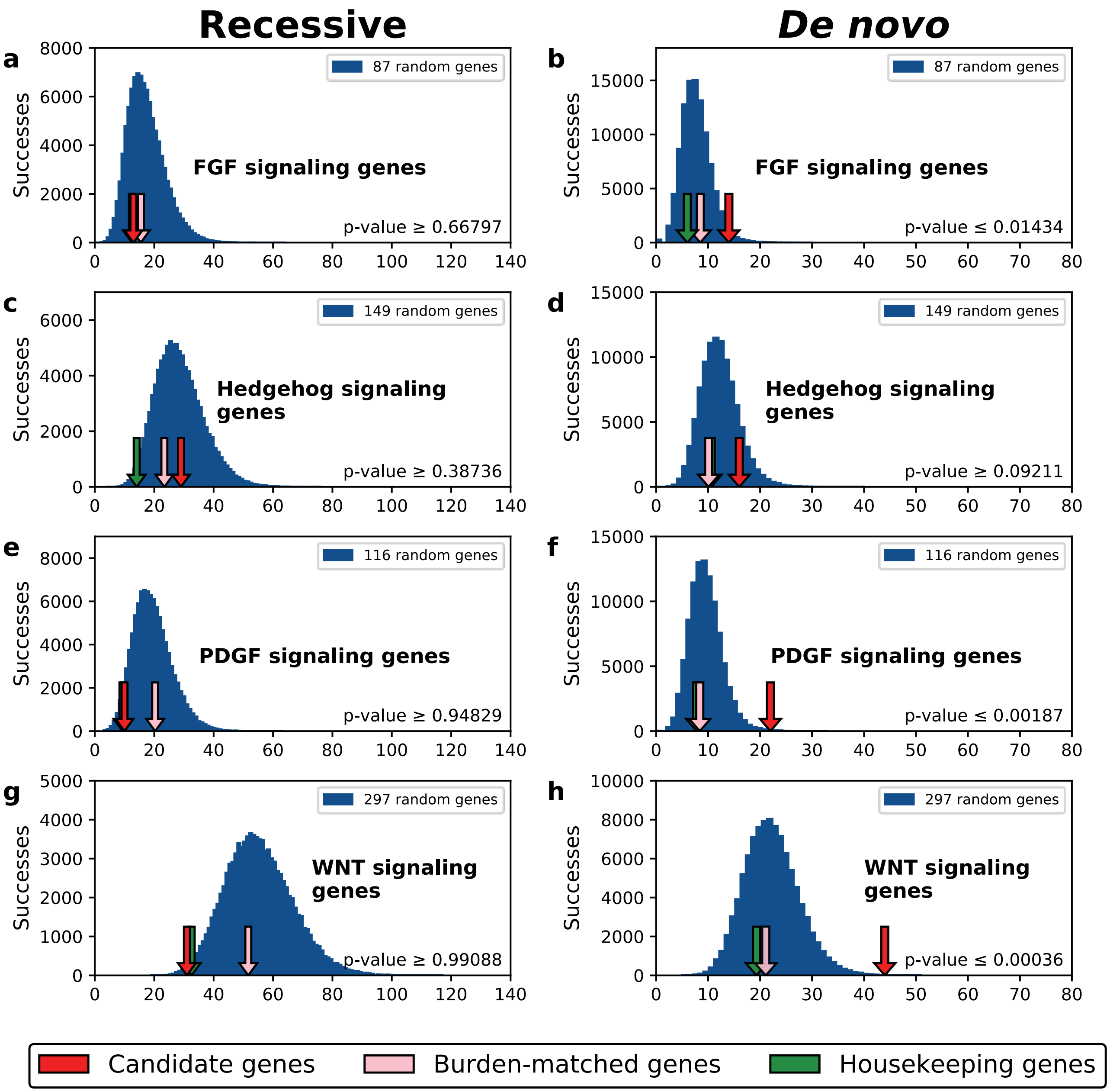

Supplementary Figure 5. Enrichment analysis in signaling pathway genes. a, c, e, g. Unlike cilia genes, signaling pathway genes showed no overall enrichment in damaging recessive genotypes. **b, f, h.** A modest enrichment for damaging *de novo* mutations in FGF (87), PDGF (116), and WNT (297) signaling pathway genes was observed in 2,391 CHD patients. **d.** The HH (149) signaling pathway did not show significant enrichment for *de novo* mutations. **e, g.** The PDGF and WNT pathways were depleted in damaging recessive genotypes.

Supplementary Note 6

To assess the expected genotype frequencies for damaging genotypes found in cilia-related genes in CHD patients, we estimated the frequency of each genotype using existing population allele frequency data. A general expectation of disease-causing genotypes (or alleles, depending on the inheritance model) is that the cumulative expected frequencies should not exceed the cumulative estimated incidence of the disorder. We find that the expected population genotype frequencies for the damaged recessive genotypes found in cilia-related genes are very rare (**Supplementary Figure 6**).

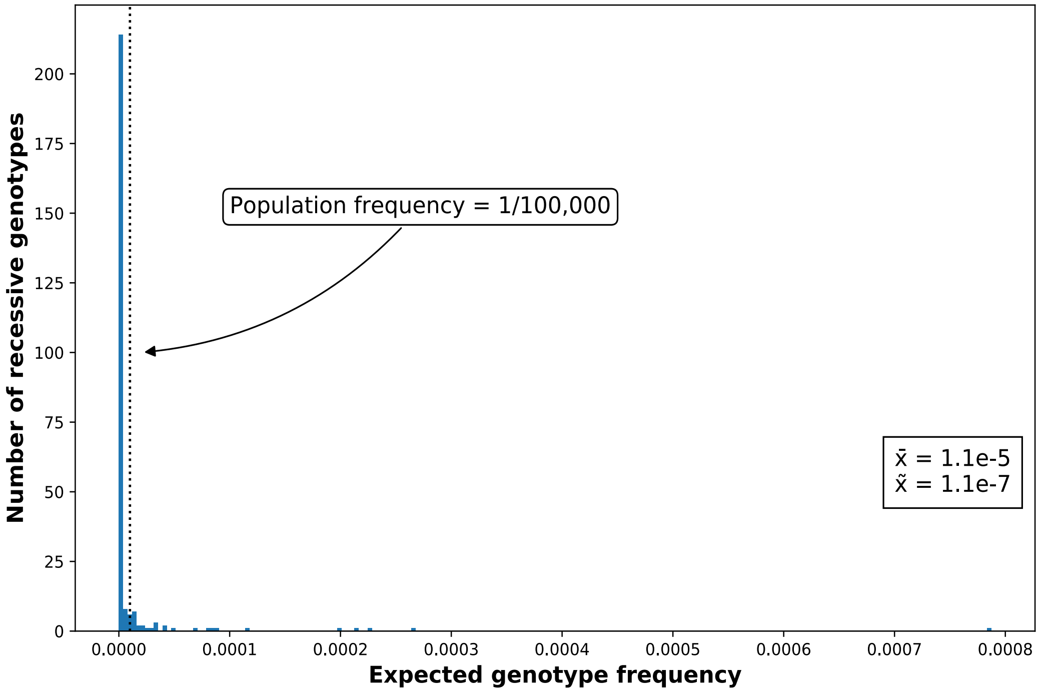

Supplementary Figure 6. Genotype frequency estimates for damaging SysCilia, Cilia, and FoxJ1 genotypes. The distribution of the expected genotype frequencies for damaging recessive genotypes found in SysCilia, Cilia, and FoxJ1 genes is shown by the histogram. Genotype frequencies were estimated as the product of the maximum allele frequency for each allele of the genotype as reported by either the gnomAD data base or by VEP annotation, which ever was higher. The dotted line indicates an estimated genotype frequency of 1/100,000. Most genotypes identified as damaging in this analysis fall below this line and are expected to be very rare in the general population, with an overall mean of 1.1×10^{-5} and a median of 1.1×10^{-7}.

Supplementary Note 7

The ancestry of individuals in a study may influence the discovery of disease-associated genetic variation. Ancestry-related effects are most pronounced in case-control analyses where mismatched cases and controls may produce false-positive disease associations which are actually caused by ancestry differences and geography-based population structure. Disease-associated variant discovery in this study was not based on a case-control strategy, but rather on the analysis of individual trios. To confirm that ancestry did not affect the discovery of damaged cilia genes, the relationship between a proband's ancestry and the presence of damaged cilia genes was analyzed by principal component analysis (PCA) (**Supplementary Figure 7**).

The ancestry of each proband was also estimated using the ADMIXTURE software package[5] and 180 reference individuals representing Africans, East Asians, Europeans, South Americans (an admixed proxy for Native Americans), and South Asians, all from the 1000 genomes project. Proband samples were processed with the reference samples in batches of 30 to avoid distortion of ancestry estimation caused by the large number of PCGC samples. Using these reference populations and a majority rule (>50%) assignment, the ancestry of the analysis cohort is estimated to be 7.4% African American, 4.4% Asian American, 81.0% European American, 3.2% Hispanic American, 2.4% South/Native American and 1.6% multiple ancestry American (see **Supplementary Data 7** for individual ancestry estimates).

a

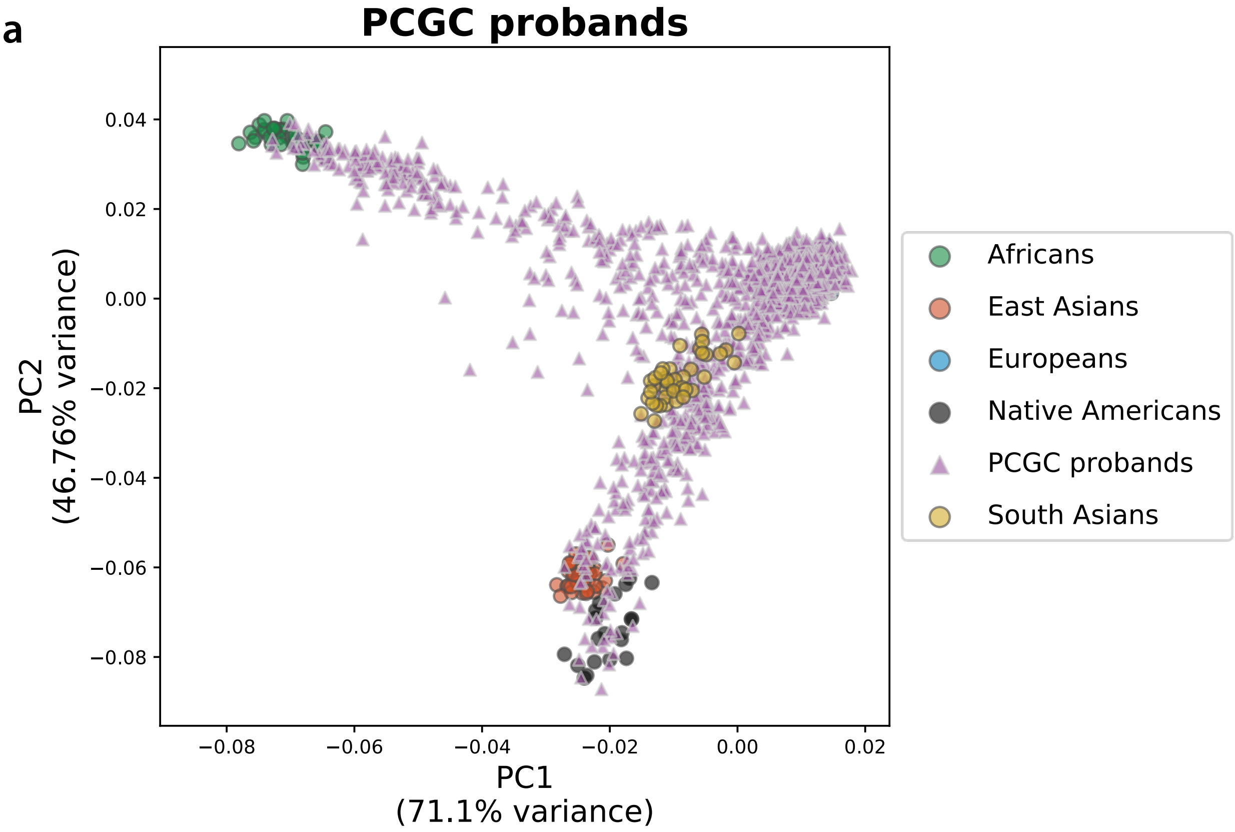
PCGC probands
0.04
0.02
0.00
−0.02
−0.04
−0.06
−0.08
PC2
(46.76% variance)
−0.08
−0.06
−0.04
−0.02
0.00
0.02
PC1
(71.1% variance)
Africans
East Asians
Europeans
Native Americans
PCGC probands
South Asians

b

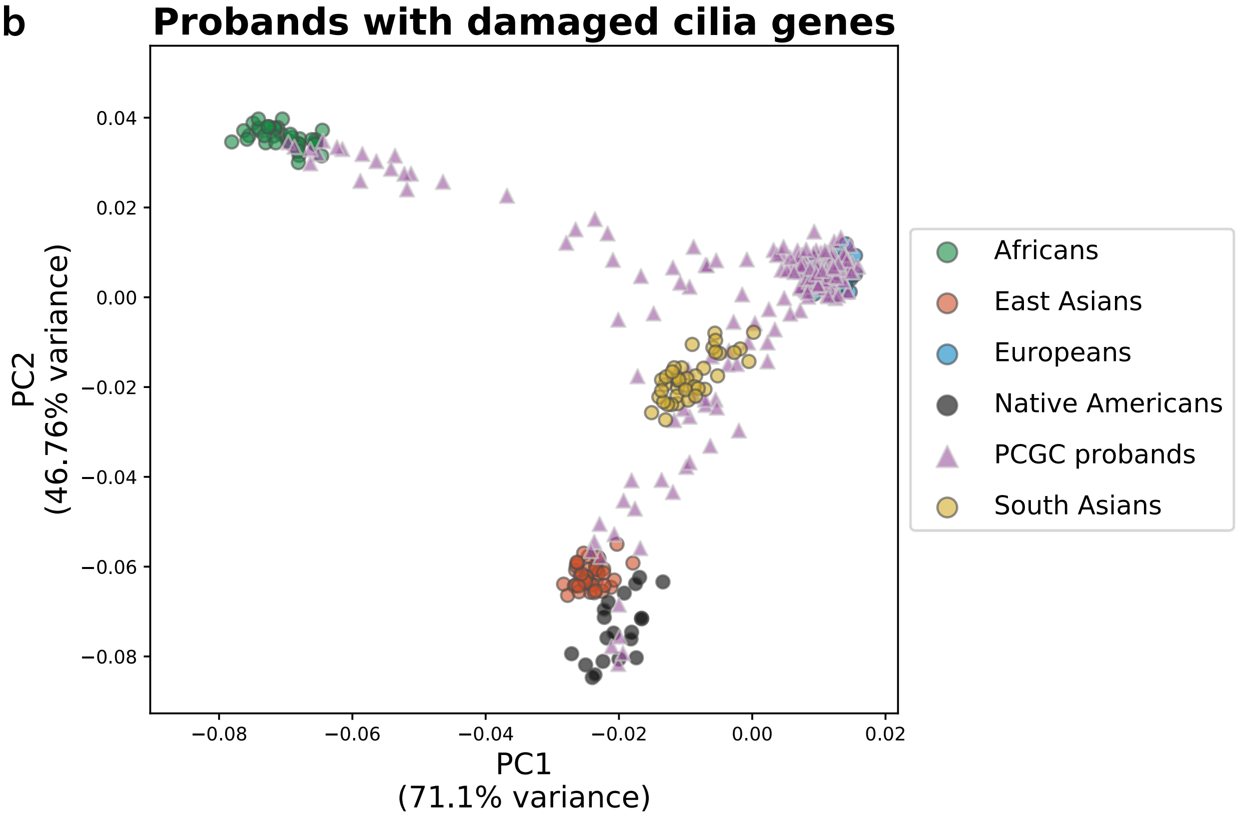
Probands with damaged cilia genes
0.04
0.02
0.00
−0.02
−0.04
−0.06
−0.08
PC2
(46.76% variance)
−0.08
−0.06
−0.04
−0.02
0.00
0.02
PC1
(71.1% variance)
Africans
East Asians
Europeans
Native Americans
PCGC probands
South Asians

Supplementary Figure 7. PCA analysis of the genetic relationships among PCGC probands. **a.** The first two principal components based on the allele-sharing genetic distances among 180 individuals from nine reference populations representing five major population groups and 2,389 PCGC probands are plotted (13,107 polymorphisms, ≤ 10% missing data, maf ≥ 0.05). Two proband samples were excluded due to missing data thresholds. Reference populations are Africans (YRI, LWK), East Asians (CHB, JPT), Europeans (CEU, GBR), South Americans (PEL), and South Asians (PJL, STU). The PCA results are highly concordant with ancestry estimates from the admixture analysis. A large majority of the cohort cluster with Europeans (upper right). In agreement with the self-reported ancestry, the PCGC probands represent ancestries from all of the five major ethnicities represented by the reference populations. Additionally, many samples have a varying degree of admixture and fall between the major reference populations, a feature that is obscured by simple categorical classification of ancestry. **b.** Panel **b** shows the Panel **a** plot except that, for PCGC samples, only PCGC probands harboring a damaging recessive cilia genotype are displayed. Probands with a damaged cilia gene are seen in all major ethnic groups in roughly the same proportions as the entire PCGC cohort. This result demonstrates that the discovery of damaged cilia genes is not restricted to, nor substantially influenced by, the ancestry of the patient.

Supplementary Tables

Supplementary Table 1. Probands with multiple damaged cilia genes

BlindedID	Number of Genes	GeneID	Phenotype
1-01184	3	DNAH6, CCDC151, NUP153	HTX
1-05270	3	GPR98, TTLL6, DNAH3	LVO
1-00620	2	DNAH7, GALNT11	HTX
1-01984	2	SYNE2, PMFBP1	LVO
1-02702	2	TUBGCP4, PARK2	HTX
1-02942	2	PCM1, IRS1	HTX
1-02990	2	HYDIN, PARK2	LVO
1-04162	2	GPR98, PCM1	CTD
1-04274	2	DRD5, TCTN2	CTD
1-05226	2	RANBP1, CTSH	CTD
1-05775	2	BBS1, MERTK	OTH
1-06817	2	PKD1, SGSM3	LVO
1-07381	2	GLI2, CEP152	OTH
1-12752	2	E2F4, MEGF8	CTD

Supplementary References

1. Manichaikul, A. *et al.* Robust relationship inference in genome-wide association studies. *Bioinformatics* **26**, 2867-73 (2010).
2. Lek, M. *et al.* Analysis of protein-coding genetic variation in 60,706 humans. *Nature* **536**, 285-91 (2016).
3. Zaidi, S. *et al. De novo* mutations in histone-modifying genes in congenital heart disease. *Nature* **498**, 220-3 (2013).
4. Jin, S.C. *et al.* Contribution of rare inherited and de novo variants in 2,871 congenital heart disease probands. *Nat Genet* **49**, 1593-1601 (2017).
5. Alexander, D.H., Novembre, J. & Lange, K. Fast model-based estimation of ancestry in unrelated individuals. *Genome Res* **19**, 1655-64 (2009).
