# Supplementary material for: De novo and recessive forms of congenital heart disease have distinct genetic and phenotypic landscapes
Source: Nat Commun. 2019 Oct 17;10:4722. doi: 10.1038/s41467-019-12582-y (PMC6797711; doi:10.1038/s41467-019-12582-y)
Supplement: Supplementary file 9 — Reporting Summary [file 41467_2019_12582_MOESM9_ESM.pdf]

nature research

Corresponding author(s): Martin Tristani-Firouzi, Mark Yandell

Last updated by author(s): 08/05/2019

Reporting Summary

Nature Research wishes to improve the reproducibility of the work that we publish. This form provides structure for consistency and transparency in reporting. For further information on Nature Research policies, see Authors & Referees and the Editorial Policy Checklist.

Statistics

For all statistical analyses, confirm that the following items are present in the figure legend, table legend, main text, or Methods section.

n/a	Confirmed	
☐	☒	The exact sample size (*n*) for each experimental group/condition, given as a discrete number and unit of measurement
☐	☒	A statement on whether measurements were taken from distinct samples or whether the same sample was measured repeatedly
☐	☒	The statistical test(s) used AND whether they are one- or two-sided<br>*Only common tests should be described solely by name; describe more complex techniques in the Methods section.*
☒	☐	A description of all covariates tested
☐	☒	A description of any assumptions or corrections, such as tests of normality and adjustment for multiple comparisons
☐	☒	A full description of the statistical parameters including central tendency (e.g. means) or other basic estimates (e.g. regression coefficient) AND variation (e.g. standard deviation) or associated estimates of uncertainty (e.g. confidence intervals)
☐	☒	For null hypothesis testing, the test statistic (e.g. *F*, *t*, *r*) with confidence intervals, effect sizes, degrees of freedom and *P* value noted<br>*Give P values as exact values whenever suitable.*
☒	☐	For Bayesian analysis, information on the choice of priors and Markov chain Monte Carlo settings
☒	☐	For hierarchical and complex designs, identification of the appropriate level for tests and full reporting of outcomes
☐	☒	Estimates of effect sizes (e.g. Cohen's *d*, Pearson's *r*), indicating how they were calculated

Our web collection on statistics for biologists contains articles on many of the points above.

Software and code

Policy information about availability of computer code

Data collection	Data collection and annotation software such as the Genome Analysis ToolKit (GATK) and the Variant Effect Predictor and their use are described in the methods and links are provided.
Data analysis	All data analysis software (Graphite, VAAST, PHEVOR, MySQL, R) and analysis details are described in the methods. Links to these software packages are provided.

For manuscripts utilizing custom algorithms or software that are central to the research but not yet described in published literature, software must be made available to editors/reviewers. We strongly encourage code deposition in a community repository (e.g. GitHub). See the Nature Research guidelines for submitting code & software for further information.

Data

Policy information about availability of data

All manuscripts must include a data availability statement. This statement should provide the following information, where applicable:

- Accession codes, unique identifiers, or web links for publicly available datasets
- A list of figures that have associated raw data
- A description of any restrictions on data availability

The sequencing data used in this analysis may be downloaded, with committee approved access, from the HeartsMart database [https://pcgc.research.cchmc.org/] or the database of Genotypes and Phenotypes (dbGaP) [https://www.ncbi.nlm.nih.gov/] (accession number: phs000571.v5.p2). Additional data files may be obtained from the authors upon request.

Field-specific reporting

Please select the one below that is the best fit for your research. If you are not sure, read the appropriate sections before making your selection.

☒ Life sciences ☐ Behavioural & social sciences ☐ Ecological, evolutionary & environmental sciences

For a reference copy of the document with all sections, see nature.com/documents/nr-reporting-summary-flat.pdf

Life sciences study design

All studies must disclose on these points even when the disclosure is negative.

Sample size	Sample size was determined by the number of sequenced trios at the time of analysis. All re-sampling experiments and sample sizes are described in the paper.
Data exclusions	We analyzed only sporadic CHD trios. Trios with known syndromic CHD (e.g. Downs, Di George syndrome, aneuploidy, etc.) were removed. A quality control step was implemented to remove trios that were outliers, that is, those that had an excessive number of de novo mutations, atypical numbers of recessive mutations, or those that had any atypical kinship co-efficients for a trio.
Replication	Re-sampling was performed using sufficient permutations to ensure that p-values obtained were stable and reproducible.
Randomization	Randomizations were performed using a large number of independent gene lists. Each list was an independent draw from the exome (18,876 genes)
Blinding	All gene lists were constructed independently and prior to any of the analyses.

Reporting for specific materials, systems and methods

We require information from authors about some types of materials, experimental systems and methods used in many studies. Here, indicate whether each material, system or method listed is relevant to your study. If you are not sure if a list item applies to your research, read the appropriate section before selecting a response.

Materials & experimental systems

n/a	Involved in the study	
☒	☐	Antibodies
☒	☐	Eukaryotic cell lines
☒	☐	Palaeontology
☒	☐	Animals and other organisms
☐	☒	Human research participants
☐	☒	Clinical data

Methods

n/a	Involved in the study	
☒	☐	ChIP-seq
☒	☐	Flow cytometry
☒	☐	MRI-based neuroimaging

Human research participants

Policy information about studies involving human research participants

Population characteristics	All trios were pediatric patients affected with CHD recruited and collected at several major U.S. hospitals. Most (~80%) had European ancestry. All patients has significant CHD phenotypes that included conotruncal defects, left ventricular outflow tract obstructions, laterality/heterotaxy, and other major CHD phenotypes.
Recruitment	Trios were recruited from major hospitals. Participation was voluntary.
Ethics oversight	This study was approved by Institutional Review Boards at all the participating institutions. All patients provided informed consent.

Note that full information on the approval of the study protocol must also be provided in the manuscript.

Clinical data

Policy information about clinical studies

All manuscripts should comply with the ICMJE guidelines for publication of clinical research and a completed CONSORT checklist must be included with all submissions.

Clinical trial registration	NCT01196182
Study protocol	https://clinicaltrials.gov/ct2/show/NCT01196182

Data collection	Ongoing. Collection occurs at several major University and regional hospitals
Outcomes	Phenotypes were assigned after examination. All phenotype assignments were completed before this analysis.
