# Supplementary material for: De novo and recessive forms of congenital heart disease have distinct genetic and phenotypic landscapes
Source: Nat Commun. 2019 Oct 17;10:4722. doi: 10.1038/s41467-019-12582-y (PMC6797711; doi:10.1038/s41467-019-12582-y)
Supplement: Supplementary file 10 — Description of Additional Supplementary Files [file 41467_2019_12582_MOESM10_ESM.pdf]

Title: Supplementary Data 1
Description: Genes included in each gene list.

Title: Supplementary Data 2
Description: Damaged SysCilia, Cilia, and FoxJ1genes in PCGC probands

Title: Supplementary Data 3
Description: Genes with damaging recessive variation in two or more probands

Title: Supplementary Data 4
Description: P-value estimates for enrichment tests

Title: Supplementary Data 5
Description: Statistics, Z-scores, and P-values for standardized distributions

Title: Supplementary Data 6
Description: Sanger confirmation of de novo and inherited compound heterozygotes

Title: Supplementary Data 7
Description: Five-component ancestry estimates for PCGC probands
